# The nature of genetic susceptibility to multiple sclerosis: constraining the possibilities

**DOI:** 10.1186/s12883-016-0575-6

**Published:** 2016-04-27

**Authors:** Douglas S. Goodin

**Affiliations:** Department of Neurology, UCSF MS Center, University of California, San Francisco, 675 Nelson Rising Lane, Suite #221D, San Francisco, CA 94158 USA

**Keywords:** Multiple sclerosis, Genetic susceptibility, Heritability, Pathogenesis, Causation, Epidemiology, Environment, Complex disease, Complex, Twin studies

## Abstract

**Background:**

Epidemiological observations regarding certain population-wide parameters (e.g., disease-prevalence, recurrence-risk in relatives, gender predilections, and the distribution of common genetic-variants) place important constraints on the possibilities for the genetic-basis underlying susceptibility to multiple sclerosis (MS).

**Methods:**

Using very broad range-estimates for the different population-wide epidemiological parameters, a mathematical model can help elucidate the nature and the magnitude of these constraints.

**Results:**

For MS no more than 8.5 % of the population can possibly be in the “genetically-susceptible” subset (defined as having a life-time MS-probability at least as high as the overall population average). Indeed, the expected MS-probability for this subset is more than 12 times that for every other person of the population who is not in this subset. Moreover, provided that those genetically susceptible persons (genotypes), who carry the well-established MS susceptibility allele (DRB1*1501), are equally or more likely to get MS than those susceptible persons, who don’t carry this allele, then at least 84 % of MS-cases must come from this “genetically susceptible” subset. Finally, because men, compared to women, are at least as likely (and possibly more likely) to be susceptible, it can be demonstrated that women are more responsive to the environmental factors that are involved in MS-pathogenesis (whatever these are) and, thus, susceptible women are more likely actually to develop MS than susceptible men. Finally, in contrast to genetic susceptibility, more than 70 % of men (and likely also women) must have an environmental experience (including all of the necessary factors), which is sufficient to produce MS in a susceptible individual.

**Conclusions:**

As a result, because of these constraints, it is possible to distinguish two classes of persons, indicating either that MS can be caused by two fundamentally different pathophysiological mechanisms or that the large majority of the population is at no risk of the developing this disease regardless of their environmental experience. Moreover, although environmental-factors would play a critical role in both mechanisms (if both exist), there is no reason to expect that these factors are the same (or even similar) between the two.

**Electronic supplementary material:**

The online version of this article (doi:10.1186/s12883-016-0575-6) contains supplementary material, which is available to authorized users.

## Background

### Introduction

Complex genetic disorders are those that are caused by the interaction of multiple genetic and environmental factors [1]. Many human diseases are examples of such disorders, including multiple sclerosis (MS) – a common neurological condition, in which recurrent immune-mediated injuries occur to the central nervous system [2, 3]. Epidemiological evidence has implicated the involvement of multiple environmental factors, including vitamin D deficiency and Epstein-Barr viral infections – see [3] for a review. Nevertheless, it is on the genetic side that most of the recent progress has come. The associations of MS with the human leukocyte antigens (HLA) on the short arm of chromosome 6 have been known for decades [2–9]. More recently, from several genome-wide association studies (GWAS) of single-nucleotide polymorphisms (SNPs) in MS, disease-associations have been identified in more than 150 different non-HLA locations scattered throughout the genome [4–7]. However, the translation of these associations into a clinically useful assessment of an individual’s disease-risk has been limited. This is due to the fact that a large proportion of the heritability for many complex diseases, including MS, remains unexplained. Indeed, in MS the 110 genes so far identified (in addition to the HLA associations) only account for only 28 % of the known heredity [4, 10]. Although a good deal of effort is currently being made to narrow this so-called “heritability gap”, it is unclear how likely these efforts are to succeed.

Much will depend upon the underlying basis of genetic susceptibility. For example, suppose that the individuals in a population exist on a continuum of susceptibility (i.e., anyone can develop the illness under the proper circumstances and a person’s individual genetic make-up only serves to make this outcome more or less likely to occur). In this case, although individuals at especially high-risk could, perhaps, be identified, the development of a sensitive and specific genetic test for susceptibility, which could be applied to the population as a whole, will likely not be possible. By contrast, if only a small segment of the population is genetically susceptible and only these susceptible individuals can develop disease, then the task of developing such a test, should, in theory, be much more likely to succeed.

The epidemiological observations regarding the various population-wide parameters such as the disease prevalence, the recurrence risk in relatives, gender predilections, and the distribution of common genetic variants (observations which have been made for years in many different parts of the world) place important constraints on the possibilities for the underlying genetic basis of susceptibility. Although applicable to any complex genetic disease, for illustrative purposes, this paper considers the nature and magnitude of these constraints as they apply to the study of MS.

### Model overview and implications

Because much of this model development is technical, an overview of the basic ideas behind the model (and their implications) is here provided for purposes of clarity. Thus, in this model, directly-measurable epidemiological data are used to estimate the likelihood that an individual from the general population is “genetically-susceptible” to getting MS. This probability is defined as *P(G)*. The definition used for this parameter is provided both below and in Table 1. In order to estimate the value of this parameter, we define certain other quantities such as the conditional probability that a “genetically-susceptible” individual will get MS {*P*(*MS*|*G*)} and the probability that an individual with MS is also “genetically-susceptible” {*P*(*MS, G*)}. By the rules of conditional probability we know that:

**Table 1:** Definitions for estimating the probability of genetic susceptibility – *P(G)*

Assume a population (*P*) of (*n*) individuals: *(i = 1,2,…,n)*		
*P(MS)*	=	The life-time probability of developing multiple sclerosis (MS) in the population
*(G*_*i*_ *)*	=	Genotype of the (i^th^) individual in the population
*P(MS│G*_*i*_ *)* = *z*_*i*_	=	Expected life-time probability of MS in the (i^th^) individual (genotype)
*(G−)*	=	The subset of “non-susceptible” individuals for whom: *P(MS│G*_*i*_ *) = 0*
*(G*_*min*_ *)*	=	The subset of “minimally susceptible” individuals for whom: 0 < *P(MS│G*_*i*_ *) < P(MS)*
*(G)*	*=*	The subset of “genetically susceptible” individuals for whom: *P(MS│G*_*i*_ *) ≥ P(MS)*
*(G*_*T*_ *–)*	*=*	the combined subset: *(G*_*min*_ *) ∪ (G–)*
*(G*_*T*_ *)*	*=*	the combined subset: *(G*_*min*_ *) ∪ (G)*
*Z, X, Y, W,V*	=	sets of: {*z*_*i*_}; in the entire population *(Z)*; in the *(G)* subset *(X)*; in the *(G−)* subset (*Y),* in the *(G*_*T*_ *)* subset *(W)*, and in the *(G*_*T*_ *–)* subset *(V)*
*P(MS│G−), P(MS│G*_*min*_ *), P(MS│G),*	=	Expected life-time probability of MS for individuals in the subsets *(G−), (G*_*min*_ *),* or *(G)*. By definition: *P(MS│G) > P(MS│G*_*min*_ *) > P(MS│G−) = 0*
*p, q*	=	*p = P(G)*; *q = P(G│MS)* = *P(G│IG*_*MS*_ *)*
*x, x’, x*_*i*_	=	*x = P(MS│G)*; *x’ = P(MS│G, IG*_*MS*_ *)*; *x*_*i*_ *= P(MS│G*_*i*_ *)* given *z*_*i*_ *ε X*
*y, y’*	=	*y = P(MS│G*_*min*_ *); y’ = P(MS│G*_*min*_ *, IG*_*MS*_ *)*
*r*	=	The largest value of *P(MS│G*_*i*_ *)* in the population
*P(MS│MZ*_*MS*_ *)*	=	The conditional life-time probability of an individual developing MS, given that their monozygotic (MZ)-twin either has or will develop MS. This is equal to the proband-wise concordance rate for MZ twins.
*P(MS│DZ*_*MS*_ *),* *P(MS│S*_*MS*_ *)*	=	The equivalent definition as for *P(MS│MZ*_*MS*_ *)* except for the individual having either a dizygotic (DZ) twin or sibling (S) with MS
*P(MS│IG*_*MS*_ *) = b*	=	*P(MS│MZ*_*MS*_ *)* adjusted for the impact of an identical genotype (IG) sharing the same childhood and intrauterine micro-environments

$$ P(G)=P\left(MS,G\right)/P\left(MS\Big|G\right) $$

Therefore, in order to estimate *P*(*G*), we just need to know (approximately) what these other two probabilities are. Fortunately, these other probabilities can be estimated from directly-observed epidemiological data. For example, it must be the case that *P*(*MS, G*) is less than the probability of MS in the population *P*(*MS*) and this probability, in turn, can be approximated by the measured prevalence of MS in the population. Also, we will define the term *P*(*MS*|*MZ*_*MS*_) as the probability that a monozygotic (MZ) twin will get MS given that their co-twin already has (or will develop) MS. This also is a measureable epidemiological parameter – the proband-wise (or case-wise) MZ-twin concordance rate for MS [10]. Moreover, because, this observed rate must be less than the concordance rate in “genetically-susceptible” MZ-twins, the observed rate can be used to approximate the term *P*(*MS*|*G*). Thus, using these two approximations for the populations of North America and northern Europe (where these two measured parameters are reasonably well-established), our estimate for *P*(*G*) becomes:

$$ P(G)\approx P(MS)/P\left(MS\Big|{MZ}_{\mathrm{MS}}\right)\approx (0.001)/(0.25)=0.004 $$

Consequently, this simple “back of the envelope” calculation suggests that the occurrence of “genetic-susceptibility” in the population must be extremely rare (~0.4 %). The present manuscript refines this calculation, providing an estimate for the maximum possible value that this probability (*G*) can take, given the uncertainties in the estimates for these two measurable parameters and given the possible relationships that these measureable parameters have to the actual parameters of interest {i.e., *P*(*MS, G*) and *P*(*MS*|*G*)}. In addition, this markedly asymmetric division of individuals into those who are “genetically-susceptible” and those who are not has important implications for the nature of genetic susceptibility to MS. Thus, in contrast to a log-normal model (in which the odds of MS are increased by each independent genetic risk factor that a person possesses), the epidemiological data strongly suggests that most (possibly all) individuals with MS come from the “genetically-susceptible” group and that the population is markedly bimodal with respect to the likelihood (risk) that individual members of the population will develop MS.

Moreover, there seem to be differences in the nature of the pathogenesis of MS between men and women. Thus, genetic risk factors are critical to each. However, because women comprise almost three quarters of the MS population, it is perhaps surprising that men are, if anything, more likely than women to be “genetically-susceptible” to getting MS. Therefore, because of this fact, the final gender distribution must be related to environmental factors (either from differences in exposure between men and women or from differences in the response by women to a given exposure). The environment is known to play a critical role in MS-pathogenesis and at least three separate environmental factors (events) are implicated. Each of these events occurs in the large majority of individuals within the population (i.e., they are population-wide environmental events). One event occurs near birth (either *in utero* or in the immediate post-natal period), another occurs during adolescence, and a third (and possibly more) occurs thereafter. Based on the observed epidemiological data, it can be shown that the basis for the final gender distribution in MS, and for the increasing proportion of women in contemporary MS cohorts, is that susceptible women are much more likely to develop MS than susceptible men under similar environmental conditions.

The remainder of this manuscript (together with the Additional file 1) is devoted to developing these ideas in a more rigorous manner.

## Methods

### Model definitions for determining genetic susceptibility in MS

1. The definitions used for establishing an upper limit for the probability of being genetically susceptible to MS in the population are listed in Table 1.

Consider a population (*P*_0_) of (*n*) individuals (*i* = 1,2,…,*n*), each with their own unique genotype (*G*_*i*_). Let the term *P*(*MS*) be defined as the expected life-time probability that a member of the population will develop MS. This probability is related to a directly-observable population parameter – the disease prevalence. Let the expected life-time probability of getting MS for a specific individual (i.e., for their unique genotype) be defined as the conditional probability *P*(*MS*|*G*_*i*_). Let (*Z*) be the set of all these individual probability values within the population. Thus:

$$ (Z)=\left\{{z}_i\right\}; $$

where: $$ \forall {G}_i\in \left({P}_0\right):{z}_i=P\left(MS\Big|{G}_i\right) $$

Further, let the population be partitioned into three mutually exclusive subsets of individuals based on their individual expected life-time probability values. These three subsets (*G*), (*G*_*min*_), and (*G*-) are defined in the following manner:

$$ \begin{array}{l}(G)=\left\{{G}_i\in \left({P}_0\right)\Big|P\left(MS\Big|{G}_i\right)\ge P(MS)\right\};\kern8em P\left(MS\Big|G\right)=x\\ {}\left({G}_{\min}\right)=\left\{{G}_i\in \left({P}_0\right)\Big|P(MS)>P\left(MS\Big|{G}_i\right)>0\right\};\kern3.5em P\left(MS\Big|{G}_{\min}\right)=y\end{array} $$

and: $$ \left(G-\right)=\left\{{G}_i\in \left({P}_0\right)\Big|P\left(MS\Big|{G}_i\right)=0\right\};\kern0.5em P\left(MS\Big|G-\right)=0 $$
By these definitions: $x\ge P(MS)>y>0$
and: $$ P(G)+P\left({G}_{\min}\right)+P\left(G-\right)=1 $$

The subset (*G*-), members of which have no chance of getting MS, will be referred to as “non-susceptible”; the subset (*G*_min_), members of which have a very small chance of getting MS. will be referred to as “minimally-susceptible”; and the subset (*G*) will be referred to as “genetically-susceptible”. Let the sets (*X*) and (*Y*) be the sets of the individual life-time probability values for members of the (*G*) and (*G*_min_) subsets respectively. Thus:

$$ (X)=\left\{{x}_i\right\}; $$

where: $$ \forall {G}_i\in (G):{x}_i=P\left(MS\Big|{G}_i\right) $$
and: $$ (Y)=\left\{{y}_i\right\}; $$
where: $$ \forall {G}_i\in \left({G}_{\min}\right):{y}_i=P\left(MS\Big|{G}_i\right) $$

Let the combined subset of all genotypes, which are not in the “genetically-susceptible” subset, be defined as:

$$ \left({G}_T-\right)=\left({G}_{\min}\right)\kern0.5em \cup \kern0.5em \left(G-\right) $$

Let the set (*V*) be the set of the individual life-time probability values for members of the (*G*_*T*_-) subset. Thus:

$$ (V)=\left\{{v}_i\right\}; $$

where: $$ \forall {G}_i\in \left({G}_T-\right):{v}_i=P\left(MS\Big|{G}_i\right) $$

And finally, let the combined subset of all genotypes, which have a non-zero probability of developing MS (*G*_*T*_), be defined as:

$$ \left({G}_T\right)=(G)\kern0.5em \cup \kern0.5em \left({G}_{\min}\right) $$

Let the set (*W*) be the set of the individual life-time probability values for members of the (*G*_*T*_) subset. Thus:

$$ (W)=\left\{{w}_i\right\}; $$

where: $$ \forall {G}_i\in \left({G}_T\right):wi=P\left(MS\Big|{G}_i\right) $$

Whether defining genetic susceptibility in this manner or creating these different categories has any utility is not known. Therefore, the value (if any) of these constructs needs to be established. Nevertheless, any population can be partitioned in this manner and such a division makes no assumptions about the underlying distribution of the individual expected life-time probability values within the population. For example, if everyone has the exact same expected life-time probability, *P*(*MS*), then everyone will belong to the subset (*G*) and the subsets (*G*_min_) and (*G*-) will be empty. If everyone has a non-zero expected life-time probability of MS, then the subset (*G*-) will be empty. If the distribution of individual expected life-time probability values {*w*_*i*_} within the (*G*_*T*_) subset is normal and centered at *P*(*MS*), then the two subsets (*G*) and (*G*_min_) will be symmetrical to each other, *P*(*G*) = *P*(*G*_min_), and each subset will have the half-normal distribution. If the distribution of individual expected life-time probability values is something else, then individuals will be assigned to the three subsets accordingly. For MS, the subset (*G*) cannot be empty; neither can it encompass the entire population [2–9].

## Results

### Estimating the probability of genetic susceptibility – *P(G)*

2. Therefore, from the definitions provided in (1) above, the proportion (*p*) of “genetically susceptible” individuals in the population (*P*_0_) is:

$$ \begin{array}{l}p=P\left(G\Big|{P}_0\right)=P(G)>0\\ {}1-p-P\left(G-\right)=P\left({G}_{\min}\right)\ge 0\\ {}1-p=P\left({G}_T-\right)>0\end{array} $$

so that: $$ P(MS)=P(G)x+P\left({G}_{\min}\right)y=px+\left\{1-p-P\left(G-\right)\right\}y $$

And also the proportion (*q*) of “genetically susceptible” individuals in the subset (*MS*) is:

$$ q=P\left(G\Big|MS\right)>0 $$

and: $$ \left(1-q\right)=P\left({G}_{\min}\Big|MS\right)\ge 0 $$

As previously, we will let the term *P*(*MS*|*MZ*_*MS*_) be defined as the conditional life-time probability that an MZ-twin will develop MS, given that his or her co-twin either already has, or will develop, MS. This probability is related to a directly-observable population parameter – the proband-wise (or case-wise) concordance rate for MZ-twins [10]. Let the purely hypothetical term, *P*(*MS*|*IG*_*MS*_), be introduced to represent the MZ-concordance rate, which has had the impact of the environment (shared by the twins) removed. Thus, this term envisions what the expected concordance rate would be if the MZ-twins, with their identical genotypes (*IG*), were to be separated at conception and to grow up independently in different environments (both intrauterine and childhood).

Let the term (*b*) be defined such that:

$$ b=P\left(MS\Big|{IG}_{\mathrm{MS}}\right) $$

Because MZ-twins are genetically identical, and because MZ-twining is thought to be non-genetic, therefore, it is assumed (see Additional file 1) that:

$$ q=P\left(G\Big|MS\right)=P\left(G\Big|{MZ}_{\mathrm{MS}}\right)=P\left(G\Big|{IG}_{\mathrm{MS}}\right) $$

Let the two quantities (*x* ') and (*y* ') be defined such that:

$$ x\hbox{'}=P\left(MS\Big|G,{IG}_{\mathrm{MS}}\right) $$

and: $$ y\hbox{'}=P\left(MS\Big|{G}_{\min },{IG}_{\mathrm{MS}}\right) $$
so that: $$ b=qx\hbox{'}+\left(1-q\right)y\hbox{'} $$

3. From the Additional file 1:

$$ x\hbox{'}\ge x\ge P(MS)>y\hbox{'}\ge y>0 $$

Moreover, because (0 < *q* ≤ 1), the final equation in (2) above can easily be rearranged to yield:

$$ 0<q=\left(b-y\hbox{'}\right)/\left(x\hbox{'}-y\hbox{'}\right)\le 1 $$

and, therefore: $$ x\hbox{'}\ge b $$

4. Let the maximum probability of MS (*r*) within the set (*X*) be defined such that:

$$ \forall {x}_i,{x}_j\in (X):{x}_j=r;\kern0.1em \mathrm{if}\kern0.3em \mathrm{it}\kern0.3em \mathrm{is}\kern0.3em \mathrm{true}\kern0.3em \mathrm{that}:\kern0.5em \forall {G}_i\in (G):{x}_i\le {x}_j $$

By definition, (*r*) is also the maximum probability within the set (*Z*). Moreover, because there must be at least one person in (*X*), for whom: *xi* ≥ *b*

therefore, it must also be the case that: *r* ≥ *b*

5. From the Additional file 1:

$$ x\hbox{'}=x+{\sigma}_X^2/x $$

or: $$ {x}^2-x\hbox{'}x+{\sigma}_X^2=0 $$
which, because of the constraint that when: ${\sigma}_X^2=0;$
then: $$ x=x\hbox{'} $$
and because, by definition: $x>0$
this has the unique solution: $x=x\hbox{'}/2+\left(\sqrt{{\left(x\hbox{'}\right)}^2-4{\sigma}_X^2}\ \right)/2$

From (3) above, under any circumstance, the following limits must apply:

$$ x\ge x\hbox{'}/2\ge b/2; $$

and: $$ {\sigma}_X^2\le {\left(x\hbox{'}\right)}^2/4 $$
Also, when: $$ \left\{x\hbox{'}=b\right\}; $$
then: $$ {\sigma}_X^2\le {b}^2/4 $$

This theoretical limit for (*σ*_*X*_^2^) is only slightly greater than the maximum possible variance for the set (*X*), which occurs for a bimodal population [11], in which:

$$ P\left\{{x}_i=b\right\}=0.5; $$

and: $$ P\left\{{x}_i=P(MS)\right\}=0.5 $$
so that: $$ b=r; $$
and: $$ E(X)=x=\left\{b+P(MS)\right\}/2 $$
and: $$ {\sigma}_X^2=E{\left({x}_i-x\right)}^2={\left\{r-P(MS)\right\}}^2/4={\left\{b-P(MS)\right\}}^2/4 $$

However, these circumstances describe a bimodal population. Therefore, if these circumstances pertain, the case for a bimodal distribution is already made. Consequently, for any unimodal distribution, (*σ*_*X*_^2^) must be lower than this upper-bound and (*x*) must be greater than this lower-bound.

6. For example, the uniform distribution is an example of unimodal distribution, which is evenly spread out [11]. The uniform distribution is defined such that:

$$ \forall {x}_i,{x}_j\in (X):P\left({x}_i\right)=P\left({x}_j\right) $$

where: $$ {\sigma}_X^2={\left\{r-P(MS)\right\}}^2/12 $$
and: $$ x=\left\{r+P(MS)\right\}/2 $$

In this circumstance, from this and from the Additional file 1:

$$ {\sigma}_X^2=x\left(x\hbox{'}-x\right)={\left\{r-P(MS)\right\}}^2/12 $$

which, when (*x* ' = *b*), can be rewritten to become:

$$ \left\{r+P(MS)\right\}b/2-{\left\{r+P(MS)\right\}}^2/4={\left\{r-P(MS)\right\}}^2/12 $$

This last expression is a quadratic in (*r*), which otherwise includes only the population parameters {*b* and *P*(*MS*)} and, thus, (*r*) in this circumstance can be estimated based on direct epidemiological observations.

7. For a population with a similar age-structure to the US, the quantity *P*(*MS*) will be between one and two times the population prevalence [2]. The prevalence of MS can be very broadly estimated to be 50–250/100,000 in northern populations [2, 3, 12–15], which yields the range-estimate for *P*(*MS*) of:

$$ 0.0005\le P(MS)\le 0.005 $$

To estimate the quantity {*b = P (MS|IG*_*MS*_*)*} requires an understanding of the impact that the shared childhood and intrauterine micro-environments have on the likelihood that MS will develop. From multiple observations, the shared childhood micro-environment seems to make little difference [15–22]. The shared intrauterine (IU) micro-environment, by contrast, may be important [13, 14, 23–29]. Thus, the Canadian data [23] regarding the recurrence risk for dizygotic (DZ) twins and siblings (S) suggests that this IU effect may be as large as:

$$ P\left(MS\Big|{DZ}_{\mathrm{MS}}\right)/P\left(MS\Big|{S}_{MS}\right)=0.054/0.029=1.86 $$

A similar disparity has been noted in a review of the available epidemiological data [14]. Nevertheless, in a recent population-based study from Sweden, DZ-twins and siblings seemed to have the same risk [13, 14].

Assuming the truth lies between these two extremes then, using a very broad range-estimate for the proband-wise concordance rates for MZ twins {i.e., *P*(*MS*|*MZ*_*MS*_)} in northern populations of between 0.15 and 0.40, inclusive [2, 3, 14, 23–29] yields the range-estimate for (*b*) of:

$$ 0.15/1.86=0.081\le \left\{b=P\left(MS\Big|{IG}_{\mathrm{MS}}\right)\right\}\le 0.4 $$

8. Substituting: {*b* = 0.081 ;  and :  *P*(*MS*) = 0.005} into the Equations from (6) above, yields the minimum possible estimate for (*x*) of:

$$ x\ge b/2\ge 0.081/2=0.0405 $$

Because: $$ P(G)=P\left(MS,G\right)/x\le 2*P\left(MS,G\right)/b\le 2*P(MS)/b $$
therefore: $$ P(G)\le 2*P(MS)/b\le 0.01/0.081=0.123 $$

Consequently, under any circumstance, the maximum possible percentage of the population (*P*_0_) that members of the (*G*) subset could comprise is 12.3 %.

A lower bound for *P*(*G*) can also be established by noting that:

$$ x\hbox{'}=P\left(MS,G\Big|{IG}_{\mathrm{MS}}\right)/P\left(G\Big|{IG}_{\mathrm{MS}}\right)=P\left(MS,G\Big|{IG}_{\mathrm{MS}}\right)/P\left(G\Big|MS\right) $$

Because: $$ b=P\left(MS\Big|{IG}_{\mathrm{MS}}\right)\ge P\left(MS,G\Big|{IG}_{\mathrm{MS}}\right); $$
and because: $$ x\hbox{'}\ge x $$
Therefore: $$ P(G)=P\left(MS,G\right)/x\ge P\left(MS,G\right)/x\hbox{'}\ge {\left\{P\left(G\Big|MS\right)\right\}}^2\Big\{P(MS)/b\Big) $$

And, using the definition of (*g*) from (Additional file 1: Table S1) as:

$$ g=P\left(G\Big|MS\right) $$

the maximum possible range for *P*(*G*) can be expressed as:

$$ \left({g}^2\right)\left\{P(MS)/b\right\}\le P(G)\le (2)\left\{P(MS)/b\right\} $$

9. Nevertheless, as noted in (5) above, this particular upper-bound is that for a bimodal population. Substituting these same values into the Equations from (6) above for the uniform distribution {at: (*x* ' = *b*)} yields the minimum (*x*) and the maximum variance of (*X*) for this situation of:

$$ r=0.121;\kern0.5em x=0.063;\kern0.5em {\sigma}_X^2=0.0011; $$

and: $$ {\sigma}_X=0.034 $$

Notably, however, the largest possible variance for any unimodal population [11], has been shown to be:

$$ {\sigma}_X^2=x\left(x\hbox{'}-x\right)={\left\{r-P(MS)\right\}}^2/9 $$

Therefore, using the same estimates for {*b* and *P*(*MS*)} as above, yields

$$ r=0.113;\kern0.5em x=0.059;\kern0.5em {\sigma}_X^2=0.0013; $$

and: $$ {\sigma}_X=0.036 $$

From (5) above, as either (*b*) increases or as (*x* ') increases relative to (*b*), both the minimum (*x*) and the maximum variance of (*X*) increase.

Despite this slightly larger upper-bound for the variance of any unimodal distribution compared to the uniform distribution, several conditions (e.g., the distribution is symmetrical, the median is equal to *x*, or the mode is equal to *x*) are sufficient to make the maximum variance be that of the uniform distribution [11]. Nevertheless, the larger estimate for (*σ*_*X*_^2^) − i.e., that for any unimodal distribution − and the smaller estimate for the minimum (*x*) will be used in the remainder of the calculations.

10. Finally, because the probability of the part cannot exceed the probability of the whole, it follows that:

$$ P(MS)/P(G)\ge P\left(MS,G\right)/P(G)=P\left(MS\Big|G\right)=x $$

Using the result from (9) above that (*x* ≥ 0.059), therefore:

$$ P(MS)/P(G)\ge x\ge 0.059 $$

With simple rearrangement, this condition means that the maximum possible estimate for the upper-bound of *P*(*G*), given any unimodal distribution of the set (*X*), is:

$$ P(G)\le P(MS)/x\le 0.005/0.059=0.085 $$

11. Recall that the subset of all genotypes that have a non-zero probability of developing MS was defined as:

$$ \left({G}_T\right)=(G)\kern0.5em \cup \kern0.5em \left({G}_{\min}\right) $$

It is noteworthy, however, that the difference in the expected life-time probability of MS between these two subsets of (*G*_*T*_) is substantial.

Thus, because: $$ P\left(MS\Big|{G}_T-\right)\le \left\{y=P\left(MS\Big|{G}_{\min}\right)\right\}<P(MS)\le 0.005 $$
and because: $$ x=P\left(MS\Big|G\right)\le 0.059 $$
Therefore: $$ x/y=P\left(MS\Big|G\right)/P\left(MS\Big|{G}_{\min}\right)>P\left(MS\Big|G\right)/P(MS)\ge 0.059/0.005=12 $$
so that: $$ x=P\left(MS\Big|G\right)>12*P(MS)>12*P\left(MS\Big|{G}_{\min}\right)\ge 12*P\left(MS\Big|{G}_T-\right) $$

In fact, by the definition of the (*G*_min_) and (*G*-) subsets, the expected probability of MS in the set (*X*) is also more than 12 times the likelihood of MS developing for every other individual member of the population who is not in the (*G*) subset.

Contrast this with the very small difference in subset means permitted for any symmetric distribution centered on *P*(*MS*). Thus, by the definition of symmetry:

$$ x-P(MS)=P(MS)-y $$

Because, by definition: $0<y<P(MS)$
Therefore, in this situation: $0<y<P(MS)\le x<2*P(MS)$

12. From (10) above, the fact that:

$$ P\left(MS\Big|G\right)=x\ge 0.059 $$

and: $$ P(G)\le 0.085 $$
and, the definition that: $0<P\left(MS\Big|{G}_{\min}\right)=y<P(MS)$

together with the extreme separation of the subset mean {*P*(*MS*|*G*)} from the means of the subsets {*P*(*MS*|*G*_min_), *P*(*MS*|*GT*−), and *P*(*MS*)}, constrain, in important ways, the possibilities for the distribution of (*Z*), which is the set of individual life-time MS probabilities in the population.

For example, the observation that no more than 8.5 % of the population can possibly be members of a unimodal subset (*G*) indicates that (*Z*) can’t have a symmetric distribution centered on *P*(*MS*). This is because, in such a circumstance:

$$ \forall {z}_i\in (Z):P\left({z}_i\in X\right)=P(G)=P\left({G}_T-\right)=P\left({z}_i\in V\right) $$

whereas, in fact: $P(G)\le 0.085<<0.915\le P\left({G}_T-\right)$

**Fig. 1:**
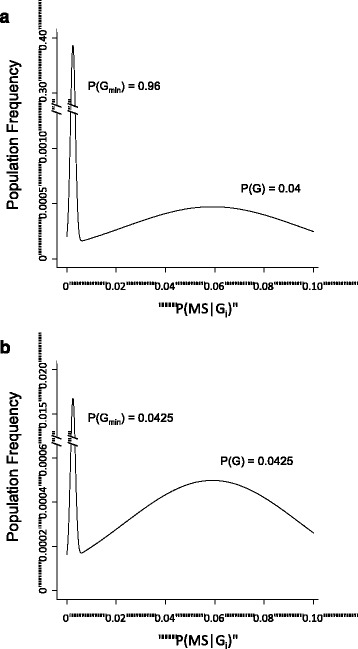
Plots of the distribution of MS-probabilities in the (*W*) set − i.e., from the combined set (*G*_*T*_) − are shown in two hypothetical circumstances, chosen to match the conditions derived from the Model. Because, as noted in the text, {*PMS*|*G*) > 12 * *P*(*MS*)}, the minimum asymmetry of the distribution is far too great to be explained by a log-normal distribution (see Additional file 1). For illustrative purposes only, the distriburtion of the MS-probabilities in both of the (*G*_*T*_) subsets are plotted as normal. However, because of the large difference in sub-subset means (see text and below), this arbitrary choice makes no difference to the final conclusions [29]. The means (μ) and standard deviations (σ) for the two distributions have been chosen to fit with the following four derived or defined relationships (see text and Additional file 1): 1. *E*(*X*) = *x* = *P*(*MS*|*G*) ≥ 0.059; 2. 0 < *y* = *P*(*MS*|*G* min) < *P*(*MS*) ≤ 0.005; 3. *P*(*MS*) = *P*(*G*)*x* + *P*(*G* min)*y* and: 4. *σ*_*X*_^2^ = *E*(*xi* − *x*)^2^ ≤ 0.0013 ; ∴ *σ*_*X*_ ≤ 0.036. Thus, for the distribution surrounding *P*(*MS*|*G*), these values have been taken to be (*μ*1 = 0.055 ; *σ*1 = 0.036) and for the distribution surrounding *P*(*MS*|*G* min)*,* they have been taken to be (*μ*2 = 0.0025 ; *σ*2 = 0.001). The total area underneath the entire (*W*) distribution (depicted) is equal to *P*(*G*_*T*_). The areas underneath each sub-distribution {i.e., splitting the two distributions at *P*(*MS*)}, are equal to *P*(*G*) and *P*(*G*_min_), respectively. For the first distribution, (*μ*_1_) is slightly less than {*x* = *P*(*MS*|*G*)} because a small portion of the left-hand tail of this distribution belongs to the (*G*_min_) subset. The first plot (panel A), considers the distribution of (*W*) in a circumstance where the distribution of MS probabilities in the combined (*G*_*T*_-) set is unlikely to be bimodal {i.e., where *P*(*G*-) = 0}. In this circumstance, the area underneath (and, thus, the height of) the curve representing the (*X*) distribution (i.e., for the “genetically susceptible” subset) depends upon the value of *P*(*MS,G*_min_). Thus: 5. when: *P*(*MS*, *G*_min_) ≈ 0; then: *P*(*G*) ≈ *P*(*MS*)/*x*; 6. when: *P*(*MS*, *G*_min_) ≈ 0.5 * *P*(*MS*); then: *P*(*G*) ≈ 0.5 * *P*(*MS*)/*x*; 7. when: *P*(*MS*, *G*_min_) ≈ *P*(*MS*); then: *P*(*G*) ≈ 0. However, because {*P*(*MS*, *G*_min_) ≤ 0.56 * *P*(*MS*)}, circumstance #7 is impossible (see Additional file 1). Panel A represents circumstance #6. By contrast, the second plot (panel B), considers the distribution of (*W*) in a circumstance where the distribution of (*Z*) is definitely bimodal. Specifically this figure considers the circumstsnce, in which: *P*(*G*−) > 0.83; and: *P*(*G*_min_) ≤ *P*(*G*). In the particular case illustrated − i.e., where *P*(*G* min) ≈ *P*(*G*) ≈ 0.0425 − the distribution is actually trimodal although the zero probability of MS for the subset (*G*-) subset is not depicted in the graph, despite the fact that this subset constitutes the large majority of the population. If the distribution for the (*G*_min_) subset were assumed to have a uniform distribution, on the plot in Panel B, the plateau of the (*G*_min_) distribution would be above the population frequency of (0.0008) and, thus, would still be clearly bimodal. This same pattern persists regradless of the actual value chosen for *P*(*G*) ≈ *P*(*G*_min_). Both panels A and B demonstrate the severly bimodal character for the (*W*) distribution that results under any circumstance. Indeed, such severe bimodality will exist, regardless of the acutal shape of the distribution of MS probabilities for members of each of the two subsets of (*G*_*T*_). This is both because of the extreme separation of the subset means and because of the very restricted range for the MS probabilities within the (*G*_min_) subset [29]

Indeed, this disparity is so large that it precludes even a roughly symmetric distribution for (*Z*), which is centered on *P*(*MS*). Moreover, the extreme separation of both the subset means {*P*(*MS*|*G*_*T*_-) and *P*(*MS*|*G*)}, as well as the means for the set (G) and the (*P*_*0*_) population {i.e., *P*(*MS*|*G*) and *P*(*MS*)}, together with the very restricted range for the MS probabilities {*v*_*i*_} in the set (*V*) − i.e., for individuals in the (*G*_*T*_-) subset − indicates that the distribution of MS probabilities for the whole population (*Z*) must be, at least, bimodal [30]. This is illustrated in Fig. 1a for the circumstances in which {*P*(*W*) ≈ *P*(*Z*) = 1}. The means and variances for the distribution used in this illustration are based on considerations developed in (9) above and, for illustrative purposes only, the two distributions of the bimodal population have been each represented as normal. Moreover, this distribution could also be trimodal − i.e., the set (*V*) could itself be bimodal − if the both of its subsets {(*G*_min_) and (*G*-)} are non-empty (e.g., Fig. 1b).

Nevertheless, there are other (unimodal) distributions, which can also be markedly asymmetric. The question, therefore, naturally arises as to how confident we are that the distribution of the odds of MS in the (*P*_*0*_) population can be distinguished from these unimodal alternatives. As noted above, the extreme separation of the sub-set means suggests that the distribution is bimodal [30]. Nevertheless, the possibility that the distribution conforms to a log-normal model needs to be considered carefully. In the first place, the log-normal distribution is both unimodal and asymmetric and, moreover, this asymmetry can be of any specified degree. In the second place, the log-normal model has considerable theoretical appeal, particularly in the setting of a complex disease such as MS, which is associated with multiple genetic risk factors [6, 7]. Thus, if these multiple risk factors are independent of each other (and there is little doubt that they are sorted independently), then (by the central limit theorem) the resulting probability distribution for the odds of MS will follow a log-normal probability density function [31]. And, indeed, Clayton and colleagues recently concluded, based on experimental evidence, that a log-normal model was appropriate for another complex genetic disease, which is comparable epidemiologically to MS – type I diabetes [31].

Similarly, in MS, a log-normal distribution of the odds could account either for the minimum asymmetry of 91.5 % in (*G*_min_) and 8.5 % in (*G*), or for an even more asymmetric split. Nevertheless, for a log-normal distribution having such a split (i.e., 91.5 %/8.5 %), the mean (*t*) for that portion of a log-normal population, which is at or above the mean for the entire distribution {i.e., *P*(*MS*)}, is more than 4-fold less than the minimum mean for the odds at *P*(*MS*|*G*)– see Additional file 1. Thus, in this circumstance:

$$ P\left(MS\Big|G\right)>4.6*t $$

As such, *P*(*MS*|*G*) cannot be the mean for this portion of a log-normal distribution (see Additional file 1). This situation is changed only slightly with even much more asymmetric splits. Thus, even when the distribution is severely asymmetric and *P*(*G*) is truly tiny (e.g., 10^−14^), there is still a more than 3-fold difference between *P*(*MS*|*G*) and the mean of that portion of the log-normal distribution, which is at or above *P*(*MS*). Indeed, even in these extreme circumstances:

$$ P\left(MS\Big|G\right)>3.1*t $$

Consequently, having a mean for the odds of getting MS in the (*G*) population {i.e., *P*(*MS*|*G*)}, which is more than 12*P(*MS*), is not compatible, under any circumstance, with the subset (*G*) simply being a part of a unimodal log-normal distribution (Additional file 1: Figure S2B).

It might be argued that such a bimodal structure implies that evidence of either strong interactions or linkage should be present – neither of which has been found. However, with so many genes involved (>150) and such a small fraction of the population being in the subset (*G*), this is not the case. Indeed, considering only susceptibility genes, it seems very likely that almost all MS patients will have a unique genotype (Additional file 1) and, empirically, this seems to be true. Thus, using the first 95 MS-associated SNPs identified in the WTCCC data set [6, 7], 105 of the genotypes (at these SNP locations) were identical in, at least, 1 pair of MS cases. Nevertheless, regardless of the basis for these apparent duplications, for all of the other 10,643 MS cases in this dataset, their genotypes (at these SNP locations) were unique. Moreover, none of these apparently duplicated genotypes bore any obvious resemblance to each other – sharing identity at only 43 (on average) and 59 (at most) of the 95 SNPs. Under such circumstances, almost certainly, there will be no linkage and no strong interactions, even if the population (*P*_*0*_) is bimodal.

13. There are two further possibilities. First, it could be that {*P*(*G* min) ≤ *P*(*G*)}. In this case, the distribution of MS probabilities within (*W*) − i.e., for members of the (*G*_*T*_) subset − could either be symmetric or not. This relationship necessarily pertains for any symmetric distribution of (*W*) because, by definition, and from the above considerations:

$$ P\left(MS\Big|{G}_T\right)=P\left(MS,{G}_T\right)/P\left({G}_T\right)=P(MS)/P\left({G}_T\right)\ge P(MS) $$

From this it follows that, any symmetric distribution for (*W*) must be centered on some probability value (*μ*) such that:

$$ x\ge \mu =PMS\left|{G}_T\right)\ge P(MS) $$

and, thus, that: $P(G)\ge P\left({G}_{\min}\right)$

However, regardless of the nature of the distribution of (*W*), if this relationship holds, then it must also be the case that:

$$ P\left(G-\right)=1-P(G)-P\left({G}_{\min}\right)\ge 1-2\ast P(G)\ge 0.83 $$

In addition, the actual values that *P*(*G*_min_) can take will depend, in part, upon value of *P*(*G*).

For example, when *P*(*G*) is at its upper-bound of:

$$ P(G)=P(MS)/x $$

then: $$ P\left({G}_{\min}\right)=0 $$

From this point, as the quantity *P*(*G*_min_) becomes larger, the quantity *P*(*G*) will have to become smaller in order to maintain the relationship:

$$ P(MS)=P(G)x+P\left({G}_{\min}\right)y $$

Therefore, we conclude that, if *P*(*G* min) ≤ *P*(*G*), then it must also be the case that the large majority of the population (*P*_0_) must be in the (*G*-) subset and that the distribution of (*Z*) is, at least, bimodal.

14. Second, it could be that: {*P*(*G*_min_) > *P*(*G*)}. In this case, the extreme separation of the subset means within (*W*) − i.e., the separation of *P*(*MS*|*G*) from *P*(*MS*|*G*_min_) − together with the very restricted range for the MS probabilities in the (*G*_min_) subset, again indicates that the distribution of {*w*_*i*_} within the set (*W*) must be bimodal [30]. This is illustrated in Fig. 1b, in which the resulting extreme bimodality is demonstrated even for the circumstance where:

$$ P\left({G}_{\min}\right)\approx P(G)\approx 0.0425 $$

Again, the means and variances for the distributions used in this illustration are based on considerations developed in (9) above. This same pattern (as illustrated) persists regradless of the value chosen for *P*(*G*) ≈ *P*(*G*_min_).

## Discussion

Using very broad range-estimates for the basic epidemiological parameters of MS-prevalence and the recurrence risk of MS in MZ-twins, this analysis indicates that no more than 8.5 % of individuals in the general population can possibly be “genetically susceptible” to developing MS as herein defined. In all likelihood, this percentage is actually much smaller [2, 3]. In addition, as demonstrated in Additional file 1, more than 43 % (and, likely, more than 84 %) of MS cases develop through this genetic pathway. Importantly, each of these estimates are based on directly-observable population parameters, which have been repeatedly verified in different parts of the world.

The implications of these conclusions are substantial. Recall that the subset (*G*_min_) is defined as consisting of only those individuals who have very low individual expected life-time probability values of more than zero but less than *P*(*MS*). By contrast, individuals in the (*G*) subset, collectively, have an expected life-time probability of MS at least 12 times the maximum possible either for that of the (*G*_min_) subset as a whole or for that of any individual member of this subset.

Consequently, even though this analysis does not assume that a group of individuals who are “genetically susceptible” is distinct from other individuals in the population, it does, in fact, establish that these two groups can be so distinguished. Thus, based on the considerations developed in (13) and (14) above, there are two possibilities.

If: $$ P\left({G}_{\min}\right)\le P(G) $$
Then: $$ P\left(G-\right)=1-P\left({G}_{\min}\right)-P(G)\ge 1-2\ast P(G)>0.83 $$

And, in this situation, the two groups are distinguished by the fact that members of the “non-susceptible” subset (*G*-), which represents the overwhelming majority of the population, are at no risk of developing MS, regardless of their environmental exposure.

Conversely, if: *P*(*G*_min_) > *P*(*G*)

Then, from (14) above, even considering only those individuals who belong to the combined (*G*_*T*_) subset, the extreme separation of the means of *P*(*MS*) and *P*(*MS|G*), together with the very narrow range of MS probabilities within the (*G*_min_) subset, requires the distribution of individual expected life-time MS probabilities in the set (*W*) to be bimodal [30] and, thus, to reflect the existence of two distinct groups of MS patients (see Fig. 1a; Additional file 1: Figure S2B). In this circumstance, the two classes of MS patients are distinguished by the fact that the MS, which develops, seems to be caused by two, fundamentally different, pathophysiological mechanisms. If the subset (*G*_min_) is, indeed, non-empty, then, in the first mechanism, MS is very improbable, the genetic contribution seems to be minor, and, thus, environmental factors are likely to be primary. By contrast, in the second mechanism, MS is comparatively much more likely to occur and the combination of both genetic and environmental events are each critical determinants of disease. Importantly, if a second group of individuals with non-zero probabilities of MS actually exists, then, despite the fact that environmental factors would be involved in both mechanisms, there is no reason to expect that the environmental events involved in the first pathway are the same as (or even similar to) those involved in the second pathway.

In most cases of MS, the genetic route seems to dominate (Additional file 1). Indeed, more than 94 % of concordant MZ-twins {i.e., individuals in the (*MS,IG*_*MS*_) sub-subset} come from the (*G*) subset (Additional file 1). However, these observations do not mean that the genetics primarily determines the disease, even in these cases. In fact, the increasing prevalence of MS world-wide [32–40], its increasing prevalence in women [32, 34, 36, 39, 40], and the change in prevalence and MZ-twin concordance based on latitude [3, 12] are better explained by differences in environmental exposure and by a women’s greater physiological responsiveness to environmental events (Additional file 1) than by any differences in genetic susceptibility between groups or regions [2, 3]. Also, given the wide disparity between the probability of developing MS between men and women, the set (*G*) must itself be bimodal (Additional file 1).

These conclusions also have important implications with regard to our ability, ultimately, to determine a person’s risk through genetic analysis. Indeed, if more than 84 % of MS occurs in the genetically susceptible population (*G*) and less than 8.5 % of the population is susceptible, it should be possible, in theory, to characterize a person’s individual risk with high sensitivity and specificity. The fact that it has proven difficult to do this so far probably relates, in part, to the fact that the genetic associations have been defined on the basis of SNPs [4–7] rather than on the basis of more extended SNP-haplotypes [41, 42].

## Conclusions

It is possible to distinguish two classes of persons in the general population, indicating either that MS can be caused by two fundamentally different pathophysiological mechanisms or that the large majority of the population is at no risk of the developing this disease regardless of their environmental experience. Moreover, although environmental-factors would play a critical role in both mechanisms (if both exist), there is no reason to expect that these factors are the same (or even similar) between the two.

The definitions for the parameters used in the model are presented in Table 1.

### Ethics approval and consent to participate

Ethical approval and consent from patients were not required because neither human subjects nor animals were used. All supporting data is available to researchers in the Additional file 1 provided.

### Consent for publication

This manuscript contains does not contain any person’s individual data.

### Availability of data and materials

All of the data contained in this manuscript is publically available.

## Additional file

Additional file 1: Contains several derivations related to the points made in the main text. It also contains 3 **Table S1** and **Figure S1**. (PDF 2039 kb)

## Abbreviations

MS: Multiple sclerosis
GWAS: Genome-wide association study
MZ: Monozygotic
DZ: Dizygotic
S: Sibling
IG: Identical genotype
