# Supplementary material for: The nature of genetic susceptibility to multiple sclerosis: constraining the possibilities
Source: BMC Neurol. 2016 Apr 27;16:56. doi: 10.1186/s12883-016-0575-6 (PMC4847201; doi:10.1186/s12883-016-0575-6)
Supplement: Additional file 1: — Contains several derivations related to the points made in the main text. It also contains 3 Table S1 and Figure S1. (PDF 2039 kb) [file 12883_2016_575_MOESM1_ESM.pdf]

Supplemental Material

Supplemental Material #1 .... Genetic Considerations

Supplemental Material #2 ...... Environmental Considerations

Supplemental Material #3 .... Unimodal *vs.* Bimodal

Defining Genetic Susceptibility in Multiple Sclerosis

Supplemental Material #1

1. Assumptions A, B, & C …………..…………………..……...……..….. p.2

2. Conditional Probability of Genetic Susceptibility – $P(G \mid MS)$ ……….. p.3

3. Variance of MS-probability within the *(G)* Subset – (σ_X^2) ……..……... p.10

4. Impact of Violating Assumption (A) ……………………...………… p.11

5. Enrichment of Genotypes …..…... p.13

6. Table S1. Additional Definitions for *Supplemental Material #1*……..... p.15

7. Table S2. MZ-twin Concordance – DRB1*1501 and Gender ………... p.16

8. Gender Specific Susceptibility (*Supplemental Material #2*) ....………... p.32

Assumptions

A. It is assumed that an MZ-twin, once they know that their co-twin either has or will develop MS, doesn't (or can't) alter their behavior in such a way as to modify their subsequent risk of MS. Thus, specifically, it is assumed that:

$$\forall G_i \in (P_0) : P(MS \mid G_i, IG_{MS}) = P(MS \mid G_i)$$

As demonstrated previously [2, 3], in the Main Text, and in section (12) below, from this assumption several conclusions follow, including the following relationships:

$$0 < P(G_T-) \leq y \leq y' < P(MS) \leq x \leq x'$$

See (11) below for a consideration of the impact that violating this assumption has on the conclusions regarding genetic susceptibility.

B. Because MZ-twinning is not thought to be genetic, it is assumed that these twins are representative of the population as a whole. Specifically, here it is assumed that:

$$P(G \mid MS) = P(G \mid MZ_{MS}) = P(G \mid IG_{MS})$$

A similar assumption effectively underlies all MZ-twin studies.

C. Let (Q) represent some genetic property that an individual may or may not have. For the purposes of determining the quantity $P(G \mid MS)$, we will further assume that, regardless of whether or not individuals from the minimally (or non-) susceptible population – i.e., the set (G_T-) – possess (Q), this fact has little (or no) influence on the likelihood that MS will develop. Certainly, for the subset (G–) there can be no such influence. Specifically, it is assumed that, for many (or most) genetic traits:

$$P(MS \mid G_T-, Q+) \approx P(MS \mid G_T-, Q-) \approx P(MS \mid G_T-)$$

Estimating the Conditional Probability of Genetic Susceptibility – *P(G│MS)*

1. Additional definitions for this section are provided in Table S1. Any partition of the population could be selected although, initially, only the partition into carriers (*HLA*+) and non-carriers (*HLA*–) of the DRB1*1501 susceptibility allele is considered.

Let the terms $(g, g_1$, and $g_2)$ be defined as:

$$g = P(G \mid MS) = P(G \mid IG_{MS})$$

$$g_1 = P(G \mid HLA+, MS) = P(G \mid HLA+, IG_{MS})$$

$$g_2 = P(G \mid HLA-, MS) = P(G \mid HLA-, IG_{MS})$$

Also, let the term (*A*) be defined as: $A = P(HLA+ \mid MS) = P(HLA+ \mid IG_{MS})$

So that: $g = Ag_1 + (1 - A)g_2$

With rearrangement, this yields: $0 \le A = (g - g_2)/(g_1 - g_2) \le 1$

As a consequence, one of three conditions must hold:

$g_1 > g > g_2$; ; $g_1 < g < g_2$; or: $g_1 = g = g_2$

2. We note that:

$$b = P(MS \mid IG_{MS}) = P(MS, G \mid IG_{MS}) + P(G_{\min} \mid IG_{MS}) * P(MS \mid G_{\min}, IG_{MS})$$

By definition, every genotype in the subset $(G_{\min}, IG_{MS})$, has an expected life-time probability (within the general population – P_0) of less than $P(MS)$. Therefore, following Assumption (A), it must also be the case that:

$$y' = P(MS \mid G_{\min}, IG_{MS}) < P(MS)$$

and, consequently: $P(G_{\min} \mid IG_{MS}) * P(MS \mid G_{\min}, IG_{MS}) < P(MS)$

so that: $P(MS, G \mid IG_{MS}) > b - P(MS)$

Using the same range-estimates for $\{b = P(MS \mid IG_{MS})\}$ and $P(MS)$ as in the Main Text:

$$P(G \mid MS, IG_{MS}) > \{b - P(MS)\} / b \geq (0.081 - 0.005) / 0.081 = 0.94$$

Thus, it must be the case that more than 94% of the subset (MS, IG_{MS}) is made up of "genetically susceptible" individuals.

Consequently, the possible range for gx' is:

$$b = P(MS \mid IG_{MS}) \geq P(MS, G \mid IG_{MS}) = gx' > (0.94)b$$

Therefore, also, the possible ranges for $(g_1 t')$ and $(g_2 s')$ are:

$$t = P(MS \mid HLA+, IG_{MS}) \geq P(MS, G \mid HLA+, IG_{MS}) = g_1 t' > \{(A - 0.06) / (A)\}t$$

and: $$s = P(MS \mid HLA-, IG_{MS}) \geq P(MS, G \mid HLA-, IG_{MS}) = g_2 s' > \{(1 - A - 0.06) / (1 - A)\}s$$

Using the ($HLA+$) partition in the IMSGC (2013) dataset [7] where: $A = 0.506$

$$t / g_1 \geq t' > 0.88 t / g_1 \quad ; \quad \text{and:} \quad s / g_2 \geq s' > 0.88 s / g_2$$

3. The available data [3-8] shows that:

$$P(MS \mid HLA+) >> P(MS \mid HLA-)$$

Moreover, the observation from (Table S2) is that:

$$P(MS \mid HLA+, MZ_{MS}) \approx P(MS \mid HLA-, MZ_{MS})$$

Nevertheless, despite these two observations, there is still no guarantee that:

$$P(MS \mid G, HLA-, MZ_{MS}) \leq P(MS \mid G, HLA+, MZ_{MS})$$

or that: $$s' = P(MS \mid G, HLA-, IG_{MS}) \leq P(MS \mid G, HLA+, IG_{MS}) = t'$$

Therefore, these two possibilities will be considered separately.

First, if: $s' \le t'$; then from (2) above: $0.88s/g_2 < s' \le t' \le t/g_1$

which, with rearrangement, yields: $g_1/g_2 < t/0.88s$

Second, if: $s' > t'$; then from (2) above: $s/g_2 \ge s' > t' > 0.88t/g_1$

so that: $g_1/g_2 > 0.88t/s$

4. Let the term (A_0) be defined as:

$$A_0 = P(HLA+)$$

Let the terms (g_{01} and g_{02}) be defined as:

$$g_{01} = P(G \mid HLA+)$$
$$g_{02} = P(G \mid HLA-)$$

As noted in the Main Text, the combined subset (G_T-) is defined as:

$$(G_T-) = (G_{\min}) \cup (G-)$$

Thus, the possible ranges for the joint probabilities can be bracketed as:

$$A_0 \ge P(HLA+, G_T-) = A_0 - P(HLA+, G) \ge A_0 - P(G)$$
$$1 - A_0 \ge P(HLA-, G_T-) = 1 - A_0 - P(HLA-, G) \ge 1 - A_0 - P(G)$$

Using these possible ranges, the conditional probabilities can then be bracketed as:

$$1 \ge 1 - g_{01} = P(G_T- \mid HLA+) \ge \{A_0 - P(G)\}/A_0$$
$$1 \ge 1 - g_{02} = P(G_T- \mid HLA-) \ge \{1 - A_0 - P(G)\}/(1 - A_0)$$

We can also re-express the conditional probability ($1 - g_1$) as:

$$1 - g_1 = P(G_T- \mid HLA+, MS) = P(MS, G_T- \mid HLA+)/P(MS \mid HLA+)$$

The numerator of the above expression for $(1 - g_1)$ can be re-expressed as:

$$P(MS, G_T- \mid HLA+) = (1 - g_{01})P(MS \mid G_T-, HLA+)$$

And the denominator as: $P(MS \mid HLA+) = (A/A_0)P(MS)$

Therefore: $1 - g_1 = \{1 - g_{01}\} * \{A_0/A\} * \{P(MS \mid G_T-, HLA+)/P(MS)\}$

The same derivation holds for the conditional probability $(1 - g_2)$ so that:

$$1 - g_2 = \{1 - g_{02}\} * \{(1 - A_0) / (1 - A)\} * \{P(MS \mid G_T-, HLA-) / P(MS)\}$$

Defining the term $(B \geq 0)$ as the ratio of these two probabilities yields:

$$B = (1 - g_1) / (1 - g_2) = \{(1 - g_{01}) / (1 - g_{02})\} * \{A_0 / (1 - A_0)\} * \{(1 - A) / A\} * \{P(MS \mid G_T-, HLA+) / P(MS \mid G_T-, HLA-)\}$$

so that: $g_1 = Bg_2 + (1 - B)$

If: $B < 1$; Then: $g_1 \geq g \geq g_2$;

and equality only holds when: $g_1 = g = g_2 = 1$

If: $B = 1$; Then: $g_1 = g = g_2$; for any value of (g) ;

and, thus, the partition is not associated with MS [2].

If: $B > 1$; Then: $g_1 \leq g \leq g_2$

and, again, equality only holds when: $g_1 = g = g_2 = 1$

As per Assumption (*C*) above, we will let (*Q*) represent some genetic property that an individual may or may not have. Because, by definition, every genotype in the subset (G_T-) has a very low (or zero) life-time probability of getting MS {i.e. less than *P*(*MS*)}, and because MS is a complex genetic disorder involving many genetic properties, it seems likely, for a single genetic property, that in many cases Assumption (*C*) will hold and, thus, that:

$$P(MS \mid G_T-, Q+) \approx P(MS \mid G_T-, Q-) \approx P(MS \mid G_T-)$$

Assumption (*C*) seems especially likely to be true for HLA-status, where there is observational data (Table S2) that:

$$P(MS \mid HLA+, MZ_{MS}) \approx P(MS \mid HLA-, MZ_{MS})$$

From this assumption, it follows that:

$$P(MS \mid G_T-, HLA+) / P(MS \mid G_T-, HLA-) \approx 1$$

Therefore, using the possible range brackets for the different probabilities established above, we can establish the possible range bracket for (B) as:

$$\{[A_0 - P(G)] / A\} * \{(1 - A) / (1 - A_0)\} \leq B \leq \{A_0 / A\} * \{(1 - A) / [1 - A_0 - P(G)]\}$$

5. Other than $P(G)$, which has been estimated in the Main Text, the term (B) in this situation, thus, depends only upon observable epidemiological quantities.

For the ($HLA+$) partition in the IMSGC dataset [7]:

$$A_0 = 0.241 \quad ; \quad A = 0.506 \quad ; \quad \text{and:} \quad P(G) \leq 0.085$$

Placing these values into the final equation in (4) above yields:

$$0.201 \leq B \leq 0.349$$

By (4) above, because the entire possible range for (B) is less than unity: $g_1 \geq g \geq g_2$

6. <u>In the circumstance where:</u> $s' \leq t'$

Using the relationship from (3) above, together with the published proband-wise concordance rates for ($HLA+$) and (HLA–) MZ-twins (Table S2), yields:

$$1 < g_1 / g_2 = B + (1 - B) / g_2 < t / 0.88s = 0.309 / \{(0.88)(0.287)\} = 1.2$$

or: $g_2 > (1 - B) / (t / 0.88s - B) \geq (1 - 0.349) / (1.2 - 0.349) = 0.76$

and: $g_1 = Bg_2 + (1 - B) > 0.92$

and, thus, from (1) above: $0.84 < \{P(G \mid MS) = g = Ag_1 + (1 - A)g_2\} < 1$

Consequently, in this scenario, at least 84% of MS patients belong to the genetically susceptible subset (G).

7. <u>In the circumstance where:</u> $s' > t'$

Using the relationship from (3) above, together with the published proband-wise concordance rates for (*HLA*+) and (*HLA*–) MZ-twins (Table S2), the same analysis as in (6) above yields:

$$g_2 < (1-B)/(t/s-B) \leq (1-0.201)/(1.08-0.201) = 0.91$$

and: $g_1 = Bg_2 + (1-B) < 0.98$

and, thus, from (1) above: $P(G \mid MS) = g = Ag_1 + (1-A)g_2 < 0.95$

In this circumstance, however, it is not possible to establish how much less than 95% $P(G \mid MS)$ can be.

8. Genetic MS can be broken down into two component parts as follows:

$$P(MS, G \mid HLA+) = P(G \mid HLA+) * P(MS \mid G, HLA+)$$

The ambiguity regarding $P(G \mid MS)$, which arises from using the (*HLA*+) partition, results from the fact that (*HLA*+)-status seems to affect the susceptibility term $\{P(G \mid HLA+)\}$ far more than the life-time probability term $\{P(MS \mid G, HLA+)\}$ in this equation [2]. Indeed, if the life-time probability term were to be increased by the inclusion of only *(HLA+)* individuals, there would be a continuing enrichment of (*HLA*+) genotypes in (*G*), which will take place when moving from the susceptible population (*G*) to the (*G, MS*) or the (*G*, IG_{MS}) population, and then, again, when moving from the (G, IG_{MS}) population to the (G, MS, IG_{MS}) population [2, 3]. To illustrate this apparent lack of a life-time probability effect in *(HLA+)* individuals, and as shown in Table S2, (*HLA*+)-status is not continuously enriched in MS populations.

Thus: $P(HLA+) << P(HLA+ \mid MS) \approx P(HLA+ \mid MS, MZ_{MS})$

By contrast, for the gender (*F*) partition, women are continuously enriched so that, in this circumstance, the condition of $(t' > s')$ almost certainly holds [2, 3].

Thus, from the study of Willer and colleagues [23]:

$$P(F) << P(F \mid MS) << P(F \mid MS, MZ_{MS})$$

Indeed, for the second step of this enrichment process, which can only be due to a life-time probability imbalance [2], these authors report that 92% (22/24) of concordant MZ-twins {i.e., the (MS, MZ_{MS}) population} were women, compared to only 66% (88/133) women in the (MZ_{MS}) population (Table S2). It is unclear what impact, if any, this observation has on the validity of Assumption (C) that:

$$P(MS \mid G_T-, F) \approx P(MS \mid G_T-, M)$$

Gender, unlike HLA-status, is not a simple genetic characteristic but, rather, is likely to reflect the execution of very different developmental programs between the sexes during embryogenesis and a continuing difference in physiology thereafter. A violation of this Assumption (C) would affect our estimate of the parameter (B) and, thus, would change our estimate for the parameter (g). Nevertheless, for the purposes of this analysis, we will also make the assumption that, in "minimally susceptible" individuals, gender has little influence on the disease-occurrence. By definition, gender can have no such influence in "non-susceptible" individuals.

9. Using the IMSGC dataset [7] for the gender (F) partition:

$$P(F) = A_0 \approx 0.5 \quad ; \quad P(F \mid MS) = A = 0.718 \quad ; \quad \text{and:} \quad P(G) \leq 0.085$$

The same analysis as above in (5–8) for the gender (F) partition, using the different value of (A), and the data for gender in Table S2, leads to the possible range for (B) of:

$$0.326 \leq B \leq 0.473 \quad ; \quad \text{so that, again:} \quad g_1 \geq g \geq g_2$$

this time, however: $t / 0.79s = 0.339 / \{(0.79)(0.067)\} = 6.40$

so that: $g_2 > (1-B) / (t / 0.79s - B) \geq (1-0.473) / (6.40 - 0.473) = 0.09$

and, therefore, that: $g_1 = Bg_2 + (1-B) > 0.57$

which yields: $0.43 < \{P(G \mid MS) = g = Ag_1 + (1-A)g_2\} < 1$

This is not as narrow a range-estimate for $P(G \mid MS)$ as that, which was derived from the $(HLA+)$ partition when $(t' \geq s')$. Nonetheless, both estimates are mutually compatible.

Estimating the Variance of MS-probability in the *(G)* Subset – (σ_X^2)

10. Suppose that, of the (n) individuals in the population, (m) of them $(k = 1, 2, ..., m)$ belong to the subset (G).

The set (X), which was defined previously, can be alternatively defined using the subscript (k) to index the (m) members of the subset (G).

Thus, the set (X) the will be defined here as:

$$(X) = \{x_k\}$$

where: $\forall G_i \in (G) : x_k = P(MS \mid G_i)$; and: $G_k = G_i$

so that: $x_k = P(MS \mid G_k) = P(MS \mid G_k, G)$

Here (x_k) represents the probability of MS in the kth individual. Even if this probability is exactly the same as another person's, (x_k) is still unique to the kth individual.

Let the variance (σ_X^2) of MS-probability in the "genetically susceptible" subset (G) be defined as:

$$\sigma_X^2 = Var(X) = E(x_k - x)^2$$

where: $P(G_k \mid G) = P(G_k) / P(G) = 1/m$

$P(G) = m/n$

$\forall \{x_k \in (X) \text{ and: } G_k \in (G)\}$; then: $P(x_k \mid X) = P(G_k \mid G)$

$$E(x_k) = \sum_{k=1}^{m} (x_k)(1/m) = x$$

and: $$E(x_k{}^2) = \sum_{k=1}^{m} (x_k{}^2)(1/m) = x^2 + \sigma_X^2$$

Using Assumption (A), the probability $\{P(MS, G_k \mid G, IG_{MS})\}$ can be broken down as follows:

$$P(MS, G_k \mid G, IG_{MS}) = P(G_k \mid G, IG_{MS}) * P(MS \mid G_k, G, IG_{MS})$$

$$= P(G_k \mid G, IG_{MS}) * P(MS \mid G_k) = P(G_k \mid G, IG_{MS}) * (x_k)$$

Where the term $\{P(G_k \mid G, IG_{MS})\}$ can be re-written as:

$$P(G_k \mid G, IG_{MS}) = P(G_k \mid G, MS) = P(G_k, G, MS) / P(MS, G)$$

$$= P(MS \mid G_k, G) * P(G_k, G) / P(MS, G)$$

$$= (x_k) * P(G_k \mid G) / P(MS \mid G) = (x_k)(1/m)/x$$

Therefore: $P(MS, G_k \mid G, IG_{MS}) = (x_k) * \{(x_k)(1/m)/(x)\} = (x_k{}^2)(1/m)/(x)$

However: $x' = P(MS \mid G, IG_{MS}) = P(MS, G \mid G, IG_{MS}) = \sum_{k=1}^{m} P(MS, G_k \mid G, IG_{MS})$

where: $\sum_{k=1}^{m} P(MS, G_k \mid G, IG_{MS}) = \sum_{k=1}^{m} (x_k{}^2)(1/m)/(x) = E(x_k{}^2)/x$

Consequently: $x' = (x^2 + \sigma_X^2)/x = x + \sigma_X^2 / x$

so that: $\sigma_X^2 = x(x' - x)$

Impact of Violating Assumption (A)

11. It is conceivable that a gene-environment interaction could take place, such that individuals, having an MZ-twin with MS, might somehow modify their behavior in such a way as to change their subsequent likelihood of getting MS. For example, a person, knowing that their MZ-twin already had MS, might engage in less "risky" behaviors (whatever this means) and thereby reduce their MS risk.

Although, such a change in behavior might impact certain genotypes more than others, the effect of such a behavioral change on the subsequent MS risk would not be expected to go in opposite directions. Thus, we would not expect for individuals in the

subset (G) to engage in "less risky" behaviors and, at the same time, for individuals in the $(G_{\min})$ subset to engage in "more risky" behaviors. Because the possibility of such a complex interaction seems so remote, it is assumed not to take place.

Therefore, as a violation of this Assumption (A), we will consider the possibility that: $(x' < x)$. If (y') is either similarly impacted or not impacted at all by the resulting behavioral change, it will still be the case that either:

$$y' < y < P(MS)$$

or: $$y \le y' < P(MS)$$

In either circumstance, both (y) and (y') have an upper-bound of $P(MS)$.

Therefore, because: $$P(MS) = P(G)x + P(G_{\min})y$$

and: $$b = qx' + (1-q)y'$$

It must be that: $$P(G)x \le P(MS)$$

and that: $$qx' > b - P(MS) > 0.081 - 0.005 = 0.076$$

Taking the ratio of these last two equations, and considering the circumstances in which: $(x' < x)$, it follows that:

$$P(G) < \{P(MS)/(b - P(MS)\} * \{qx'/x\} < P(MS)/(b - P(MS)$$

so that: $$P(G) < P(MS)/(b - P(MS) < 0.005/0.076 = 0.066$$

Consequently, if Assumption (A) were to be violated in this manner {i.e., under conditions where $(x' < x)$}, the resulting estimate for $P(G)$ would actually be less than when Assumption (A) is true.

If individuals engaged in less "risky" behavior but the impact of this behavioral change continued to result in: $(x' \ge x)$, then the estimate for $P(G)$ presented in the Main Text would still hold. By contrast, if individuals systematically engaged in more "risky" behavior (whatever this means) upon learning that their twin had MS, and if this behavioral change increased their subsequent MS-risk, then $P(G)$ could not be reliably estimated from the epidemiological observations.

Enrichment of Genotypes with Greater Life-time Probabilities of MS

12. *In the MS Population:* Let the subset (G) be partitioned into two mutually exclusive sub-subsets ($G1$) and ($G2$) as follows:

$$(G1) = \{G_i \in (G) \mid P(MS \mid G_i) \geq x\}$$

$$(G2) = \{G_i \in (G) \mid P(MS \mid G_i) < x\}$$

so that: $P(G1) + P(G2) = P(G)$

Let the proportion (p_1) of the set (G) that are in the subset ($G1$) be defined as:

$$p_1 = P(G1 \mid G) = P(G1) / P(G)$$

and let: $q_1 = P(G1 \mid MS, G)$

Thus: $q_1 = P(G, G1, MS) / (P(MS, G) = P(G, G1) * P(MS \mid G1) / P(MS, G)$

$$= p_1 * \{P(MS \mid G1) / P(MS \mid G)\}$$

By definition: $P(MS \mid G1) \geq P(MS \mid G)$

Therefore: $q_1 = p_1 * \{P(MS \mid G1) / P(MS \mid G)\} \geq p_1$

Consequently, then the genotypes within ($G1$) will be over-represented (enriched) in the subset of patients who have already developed MS compared to the (G) population as a whole. Similarly, for any subset of genotypes, from within the population, which has a greater life-time probability of MS than some other subset from within the population, a comparable proof establishes that, such an enrichment must also occur for the former subset relative to the latter.

13. *In the IG_{MS} Population:* Suppose that, of the (m) individuals in the (G) subset, (m_1) of them ($j_1 = 1, 2, \ldots, m_1$) belong to ($G1$) and (m_2) of them ($j_2 = 1, 2, \ldots, m_2$) belong to ($G2$). As was done previously, these designations (subscripts) will be used to index members of the subsets ($G1$) and ($G2$).

If Assumption (A) holds, then:

$$P(MS, G_{j1} \mid IG_{MS}, G) = P(G_{j1} \mid MS, G) * P(MS \mid G_{j1})$$

Because, by the definition of $\{P(MS \mid G_{j1})\}$ from above:

$$\forall G_{j1} \in (G1): P(MS \mid G_{j1}) \geq P(MS \mid G) = x$$

Therefore: $$P(MS, G1 \mid MS, G) = \sum_{j1=1}^{m1} P(MS, G_{j1} \mid MS, G) \geq q_1 * x$$

and similarly: $$P(MS, G2 \mid MS, G) = \sum_{j2=1}^{m2} P(MS, G_{j2} \mid MS, G) < (1 - q_1) * x \quad ; \quad \{\text{if: } q_1 < 1\}$$

However: $$x' = P(MS, G1 \mid G, IG_{MS}) + P(MS, G2 \mid G, IG_{MS})$$

so that: $$P(MS, G1 \mid MS, G) > x' - (1 - q_1) * x = (q_1 * x) + (x' - x)$$

Taking this last equation, together with the result that:

$$P(MS, G1 \mid MS, G) \geq (q_1 * x)$$

Leads to the conclusion that: $x' - x > 0 \quad ; \quad \{\text{if: } q_1 < 1\}$

Also: $x' - x = 0 \quad ; \quad \{\text{if: } q_1 = 1\}$

And, thus: $P(MS \mid G, IG_{MS}) = x' \geq x = P(MS \mid G)$

If Assumption (A) holds, a parrallel argument establishes that the same relationship will result for any subset of the set (G_T). Thus, considering any subset (S_0) of the set (G_T), if Assumpttion (A) holds, it will be the case that:

$$P(MS \mid S_0, IG_{MS}) \geq P(MS \mid S_0)$$

Table S1. Additional Definitions for *Supplemental Material #1*

P_0	=	The entire population (*non-selected*)
$HLA+$	=	Carriers of the DRB1*1501 susceptibility allele
$HLA-$	=	Non-carriers of the DRB1*1501 susceptibility allele
F	=	women
M	=	men
g	=	$P(G \mid MS) = P(G \mid IG_{MS}) = P(G \mid MZ_{MS})$
g_1	=	$P(G \mid HLA+, MS) = P(G \mid HLA+, IG_{MS}) = P(G \mid HLA+, MZ_{MS})$
g_2	=	$P(G \mid HLA-, MS) = P(G \mid HLA-, IG_{MS}) = P(G \mid HLA-, MZ_{MS})$
g_{01}	=	$P(G \mid HLA+)$
g_{02}	=	$P(G \mid HLA-)$
A	=	$P(HLA+ \mid MS) = P(HLA+ \mid IG_{MS}) = P(HLA+ \mid MZ_{MS})$
A_0	=	$P(HLA+)$
t	=	$P(MS \mid HLA+, IG_{MS})$
s	=	$P(MS \mid HLA-, IG_{MS})$
t'	=	$P(MS \mid G, HLA+, IG_{MS})$
s'	=	$P(MS \mid G, HLA-, IG_{MS})$
B	=	$(1 - g_1) / (1 - g_2)$
x_i	=	$P(MS \mid G_i)$; for all $\{G_i\}$ in the subset (G)
X	=	the set of: $\{x_i\}$
x	=	mean of the set (X) ; $x = P(MS \mid G) = E(X) = E(x_i)$
σ_X^2	=	variance of the set (X) ; $\sigma_X^2 = Var(X) = E(x_i - x)^2$

Table S2. MZ-twin Concordance by DRB1*1501 Carrier Status and by Gender*

	(HLA+)	***(HLA–)***	**Totals**
**Concordant for MS (C)**	9	11	20
**Discordant for MS (D)**	31	42	73
**Totals**	40	53	93
**Pair-wise Concordance**	9/40 = 0.225	11/53 = 0.207	20/93 = 0.215
**Proband-wise Concordance**	0.309	0.287	0.297
	Women	***Men***	**Totals**
**Concordant for MS (C)**	22	2	24
**Discordant for MS (D)**	66	43	109
**Totals**	88	45	133
**Pair-wise Concordance**	22/88 = 0.250	2/45 = 0.044	24/133 = 0.181
**Proband-wise Concordance**	0.339	0.067	0.253

* *(HLA+)* = DRB1*1501 carriers; *(HLA–)* = DRB1*1501 non-carriers
Data from Willer et al. [23]
Pair-wise concordance calculated as: C/(C+D)

Proband-wise (case-wise) concordance calculated as: 2C/(2C+D)

– adjusted [10] for the double ascertainments (13/24 = 54%) in the study of Willer et al. [23].

Proband-wise concordances = $P(MS \mid F, MZ_{MS})$, $P(MS \mid M, MZ_{MS})$, & $P(MS \mid MZ_{MS})$

There were 19,938 MS-paitents in the CCGPSMS database for the study of

Willer et al. [23]. Of these, 14,081 were women so that: $P(F \mid MS) = 70.6\%$

Defining Environmental Susceptibility in Multiple Sclerosis

Supplemental Material #2

1. Assumptions D, E, F, G & H…………..……..………..…….……...….. p.2

2. Environmental Considerations ….…………………………………......…. p.4

3. Responses to Environmental Factors ….……………………......………….. p.5

4. The Increasing Prevalence of MS ……………………………………… p.9

5. Establishing Plausible Parameter Ranges …......................................…..... p.11

6. Gender Specific Hazard Rates and Thresholds …………...……......….... p.12

7. Gender Specific Exposures …………………………………...…...….... p.14

8. Gender Specific Susceptibility ..…... p.15

9. Table S3 – Additional Definitions for: *Supplemental Material #2* ..….... p.16

10. Figure S1 – Environmental Responses …...…………..…….……....….... p.17

Assumptions

D. We define (E) as the set of all environmental experiences, which are sufficient to cause MS in at least one "genetically susceptible" individual. The set $(E–)$ includes all environmental experiences, which are not in (E). The set (G_T-) are individuals not in (G) – see Main Text.

From previous considerations [2], it seems that: $P(MS \mid E-, G_T-) \approx 0$

Moreover, from *Supplemental Material #1* it seems that: $0.84 \leq P(G \mid MS) < 1$

so that: $P(MS, G) \approx P(MS)$

Therefore, it is here assumed that: $P(MS, G, E) \approx P(MS)$

E. Because the genetics of MS in Canada [38] are unlikely to have changed substantially between the two time-periods (i.e., 35 years, or 1-2 generations; see Table S3), the terms: $\{P(G);\ P(G \mid F);\ \text{and: } P(G \mid M)\}$ are assumed to have remained constant over this interval. Because it is also likely that the proportion of men and women in the population (P_0) has not changed substantially, therefore, it will also be the case that:

$$P(G,F) = P(F) * P(G \mid F) \approx 0.5 * P(G \mid F)$$

and: $$P(G,M) = P(M) * P(G \mid M) \approx 0.5 * P(G \mid M)$$

have not changed over the interval.

Moreover, it is assumed that any change in disease prevalence in Canada over this interval will accurately reflect the change in life-time probability of MS over the interval. Specifically, it is here assumed that:

$$\text{(MS-Prevalence - 1941-1945)/(MS-Prevalence - 1976-1980)} = P(MS)_1 / P(MS)_2$$

F. The hazard-rate functions for developing MS at different environmental exposures (u) in susceptible men $\{h(u)\}$ and women $\{g(u)\}$ are assumed to be proportional such that:

$$g(u) = R * h(u) \ ; \quad \text{where: } u = P(E)$$

G. For the purpose of deternmining the plausible parameter ranges, it is assumed that:

$$P(MS \mid F, MZ_{MS}) / P(MS \mid MZ_{MS}) \approx t / b$$

and that: $$P(MS \mid M, MZ_{MS}) / P(MS \mid MZ_{MS}) \approx s / b$$

This assumption suggests that, when converting from (MZ_{MS}) to (IG_{MS}), the impact of the shared intrauterine (*IU*) and childhood environments is proportionately similar for men and women. Indeed, observationally, this seems to be the case. Thus, although the sample sizes are very small, the obsevational data [23] is that:

$$P(MS \mid F, DZ_{MS}) / P(MS \mid F, S_{MS}) = 0.051/0.039 = 1.31$$

$$P(MS \mid M, DZ_{MS}) / P(MS \mid M, S_{MS}) = 0.057/0.019 = 3.0$$

If anything, this data might be taken to support the notion that men experience a greater impact from the shared *IU* environment than do women (a circumstance that only magnifies the differences between the two sexes). Nevertheless, likely, these data reflect no difference in the impact of the shared *IU* environment between men and women.

(NB: Equal hazard from all environmental sources has been used for Figure S1)

H. For the purpose determining the gender-specific susceptibility, it is assumed that:

$$P(MS, F \mid G, IG_{MS}) / P(MS, F \mid G) \approx P(MS \mid G, IG_{MS}) / P(MS \mid G)$$

$$P(MS, M \mid G, IG_{MS}) / P(MS, M \mid G) \approx P(MS \mid G, IG_{MS}) / P(MS \mid G)$$

This assumption suggests that impact of having an identical twin with MS on a person's risk of developing MS is, proportionately, the same for "genetically susceptible" men and women.

Environmental Considerations

1. New definitions pertaining specifically to *Supplemental Material #2* are listed in Table S3. From Assumption (D), it follows that:

$$P(G,F) * PMS,E \mid G,F) \approx P(MS,F) = P(MS) * P(F \mid MS)$$

and: $$P(G,M) * PMS,E \mid G,M) \approx P(MS,M) = P(MS) * P(M \mid MS)$$

From Assumption (E) and defining the parameter (C) to represent the change in MS-prevalence between the two time periods (indicated by subscripts 1 and 2 – see Table S3), it follows that:

$$P(MS)_1 = C * P(MS)_2$$

and that this increase reflects a change in the amount of environmental exposure (sufficient to produce MS), which has taken place for the population over this interval of time.

Therefore: $$P(MS)_1 * P(F \mid MS)_1 = C * P(MS)_2 * P(F \mid MS)_1$$

and: $$P(MS, E \mid G, F)_1 \approx C * P(MS)_2 * P(F \mid MS)_1 / P(G, F)$$

2. In Canada, the sex-ratio in MS patients $\{i.e., P(F \mid MS) / P(M \mid MS)\}$ has increased [40] from 2.2 during the first time-period (i.e., 1941-1945) to become 3.2 during the second time-period (i.e., 1976-1980). From Assumption (E), and defining the probability of environmental MS in "genetically susceptible" women (Zw) and men (Zm), it follows that:

$$Zw_2 = P(MS, E \mid G, F)_2 \approx P(F \mid MS)_2 * P(MS)_2 / P(G, F)$$

and: $$Zw_1 = P(MS, E \mid G, F)_1 \approx P(F \mid MS)_1 * C * P(MS)_2 / P(G, F)$$

$$= \{P(F \mid MS)_1 / P(F \mid MS)_2\} * C * (Zw_2)$$

Similarly, for men:

$$Zm_2 = P(MS, E \mid G, M)_2 \approx P(M \mid MS)_2 * P(MS)_2 / P(G, M)$$

and: $$Zm_1 = P(MS, E \mid G, M)_1 \approx P(M \mid MS)_1 * C * P(MS)_2 / P(G, M)$$

$$= \{P(M \mid MS)_1 / P(M \mid MS)_2\} * C * (Zm_2)$$

Responses to Environmental Factors

3. The set (E) includes all environmental experiences, which are sufficient to cause MS in at least one "genetically susceptible" individual. The exposure level (u) was defined as the probability that an individual in the population will experience an exposure (E). From standard Survival Analysis methods, we define the cumulative survival $\{S(u)\}$ and failure $\{F(u)\}$ functions as well as the hazard-rate functions in both men $\{h(u)\}$ and women $\{g(u)\}$.

Thus, from Assumption (F): $g(u) = R * h(u)$; where: $u = P(E)$

Let $\{H(u)\}$ be defined as the definite integral of the hazard-function $\{h(u)\}$ from a (u) level of exposure to a (0) level. The (u) units can then be transformed into (a) units such that:

$$a = H(u); \quad \text{and:} \quad da = h(u)du$$

The (a) units are arbitrary and, therefore, "1 unit" of exposure will be defined such that:

$$a_2 - a_1 = 1$$

Thus, for men: $$\ln[S(u)] = -\int_0^u h(u)\,du = -\int_0^a da = -a$$

And, for women: $$\ln[S(u)] = -\int_0^u g(u)\,du = -\int_0^a R\,da = -Ra$$

Taking the anti-log of both sides of this last equation yields:

$$S(u) = e^{-Ra}$$

so that: $F(u) = 1 - e^{-Ra}$; by definition, for men: $R = 1$

4. The cumulative probability of failure in susceptible individuals (i.e., of developing MS), in the circumstance where everyone fails given a sufficient environmental exposure (*E*) – {i.e., when $P(MS \mid E,G) = 1$} – is:

$$F(u) = P(MS,E \mid G) = P(MS \mid E,G) * P(E \mid G) = P(E \mid G)$$

Therefore, for women in this circumstance:

$$F(u) = 1 - S(u) = 1 - e^{-Ra} = P(E \mid G,F)$$

and for men:

$$F(u) = 1 - S(u) = 1 - e^{-a} = P(E \mid G,M)$$

Nevertheless, unlike true survival (where everyone fails given sufficient time), the probability of developing MS may not become 100% as the probability of a sufficient environmental exposure increases to $\{P(E) = 1\}$. In addition, men and women may not approach the same limiting value for the probability of developing MS. Finally, the level of environmental exposure at which the development of MS becomes possible (i.e., the threshold) does not need to occur at a zero level of exposure for both men and women; neither does the threshold need to be the same for men as it is for women. Nevertheless, by the definition of the set (*E*):

$$\text{If: } P(E) > 0 \text{ ; then: } P(MS) > 0$$

Therefore, defining the threshold in men (λm) and women (λw), defining the threshold difference between women and men ($\lambda = \lambda w - \lambda m$), defning the probability of environmental MS in men (*Zm*) and women (*Zw*), defining the maximum probability of developing environmental MS in both "genetically susceptible" men (*c*) and women (*d*), and, finally, from Assumption (D), it follows that:

$$P(MS \mid G,F) \approx P(MS,E \mid G,F) = Zw = d * \{1 - e^{-R(a - \lambda m - \lambda)}\}$$

$$P(MS \mid G,M) \approx P(MS,E \mid G,M) = Zm = c * \{1 - e^{-(a - \lambda m)}\}$$

Moreover, following the logic of (5) and (10) in the Main Text, it follows that:

$$t'/1.4 \le P(MS \mid G,F) \le t'$$

and that: $$s'/1.4 \le P(MS \mid G,M) \le s'$$

If (c) and (d) are equal, then men and women approach the same limiting probability of developing MS. If (c) and (d) are both 1.0, then, as for true survival, everyone ultimately fails. If the threshold in women (λw) is greater than that in men (λm), then the difference in threshold (λ) will be positive. Because Assumption (F) leads to exponential response curves, any two points determine each curve uniquely.

5. Also from (2) in *Supplemental Material #1*:

$$t = P(MS \mid F,\ IG_{MS}) \geq g_1 t' \geq \{(A - 0.06) / A\} t$$

and: $$s = P(MS \mid M, IG_{MS}) \geq g_2 s' \geq \{(1 - A - 0.06) / (1 - A)\} t$$

Thus, in the case of women, the possible range for ($g_1 t'$) is:

$$t \geq g_1 t' \geq \{(0.718 - 0.06) / 0.718\} t = 0.916t$$

or: $$(t / g_1) \geq t' \geq 0.916 * (t / g_1)$$

Similarly, in the case of men, the possible range for ($g_2 s'$) is:

$$s \geq g_2 s' \geq \{(0.282 - 0.06) / 0.282\} s = 0.787s$$

or: $$(s / g_2) \geq s' \geq 0.787 * (s / g_2)$$

From Assumption (G): $$P(MS \mid F, MZ_{MS}) / P(MS \mid MZ_{MS}) \approx t / b$$

and: $$P(MS \mid M, MZ_{MS}) / P(MS \mid MZ_{MS}) \approx s / b$$

Then, from Table S2: $$t = (t / b)*b \approx (0.339 / 0.253)*b = 1.340b$$

$$s = (s / b)*b \approx (0.067 / 0.253)*b = 0.265b$$

And, using the sex ratio data from Orton an colleagues [38], which is provided in (2) above, yields:

$$P(F \mid MS)_2 = 3.2 / (1 + 3.2) = 0.762$$

$$P(F \mid MS)_1 = 2.2 / (1 + 2.2) = 0.688$$

$$P(F \mid MS)_1 / P(F \mid MS)_2 = 0.688 / 0.762 = 0.902$$

$$P(M \mid MS)_1 / P(M \mid MS)_2 = 0.312 / 0.238 = 1.313$$

6. From the equations developed in (4) above using the transformed exposure units, four relationships follow:

1. $Zw_2 = d*\{1 - e^{-R(a1+1-\lambda m-\lambda)}\}$
2. $Zm_2 = c*\{1 - e^{-(a1+1-\lambda m)}\}$
3. $Zw_1 = d*\{1 - e^{-R(a1-\lambda m-\lambda)}\}$
4. $Zm_1 = c*\{1 - e^{-(a1-\lambda m)}\}$

7. From the relationships for (t), (t'), (s), and (s') developed in (4) and (5) above, from the gender relationships described in (5) above, and using the equations for (Zw_1) and (Zm_1) from (2) above, the following possible ranges can be established:

1. $0.916*(1.340b)/g_1 = 0.1228*(b/g_1) \leq t'_2 \leq 1.340*(b/g_1)$
2. $0.788*(0.265b)/g_2 = 0.209*(b/g_2) \leq s'_2 \leq 0.265*(b/g_2)$
3. $0.902*C*(1.222)*(b/g_1) \leq t'_1 \leq 0.902*C*1.340*(b/g_1)$
4. $1.313*C*(0.209)*(b/g_2) \leq s'_1 \leq 1.313*C*0.265*(b/g_2)$

or, equivalently, using the relationships from (4) above:

1. $(1.228)*(b/g_1)/1.4 = (0.896)*(b/g_1) \leq Zw_2 \leq 1.340*(b/g_1)$
2. $(0.209)*(b/g_2)/1.4 = (0.152)*(b/g_2) \leq Zm_2 \leq 0.265*(b/g_2)$
3. $(1.108)*C*(b/g_1)/1.4 = (0.809)*(b/g_1) \leq Zw_1 \leq 1.209*C*(b/g_1)$
4. $(0.274)*C*(b/g_2)/1.4 = (0.200)*(b/g_2) \leq Zm_1 \leq 0.348*C*(b/g_2)$

These relationships are illustrated in Figure S1.

The Increasing Prevalence of MS

9. MS-prevalence is thought to be increasing [32-40]. Nevertheless, it seems unlikely that, over the interval of 35 years, it could have increased in Canada by four-fold or more [3]. Consequently, it will be assumed that: $(C > 0.25)$. Re-arrangement of Equations #1 and #3 in (6) above yields:

$$(Zw_2 - d)/d = -\{e^{-R(a1-\lambda m-\lambda)}\} * e^{-R}$$

and: $$(Zw_1 - d)/d = -\{e^{-R(a1-\lambda m-\lambda)}\}$$

Dividing these two Equations yields:

$$(Zw_2 - d)/(Zw_1 - d) = e^{-R}$$

or: $$d = \{Zw_2 - Zw_1 * e^{-R}\}/(1 - e^{-R})$$

and: $$R = -\ln\{(Zw_2 - d)/(Zw_1 - d)\}$$

Substituting for (Zw_1) from the Equations in (2) above, yields:

$$d = (Zw_2)\{1 - [P(F \mid MS)_1 / P(F \mid MS)_2]*C * e^{-R}\} / (1 - e^{-R})$$

A similar derivation using Equations #2 and #4 from (6) above yields:

$$(Zm_2 - c)/(Zm_1 - c) = e^{-1}$$

or: $$c = \{Zm_2 - Zm_1 * e^{-1}\}/(1 - e^{-1})$$

Substituting for (Zm_1) from the Equations in (2) above, yields:

and: $$c = (Zm_2)\{1 - [P(M \mid MS)_1 / P(M \mid MS)_2]\}*C * e^{-1} / (1 - e^{-1})$$

10. Because (c) is $\{P(MS \mid E, G, M)\}$, which represents the maximum possible probability for $\{P(MS, E \mid G, M)\}$, therefore:

$$Zm_2 < c = [Zm_2 - (Zm_1)e^{-1}] / (1 - e^{-1})$$

Again substituting for (Zm_1) from the Equations in (2) above, yields:

$$Zm_2 / (1 - e^{-1}) - Zm_2 \geq [P(M \mid MS)_1 / P(M \mid MS)_2](Zm_2)Ce^{-1} / (1 - e^{-1})$$

Dividing through by (Zm_2), rearranging, and using the observed sex ratio change in Canada from (2) and (5) above, this becomes:

$$C < \{1/(1 - e^{-1}) - 1\} / \{[P(M \mid MS)_1 / P(M \mid MS)_2]e^{-1} / (1 - e^{-1})\} = 0.76$$

Thus, the possible range for (C) is: $0.25 < C < 0.76$

Consequently, $P(MS)$ must have increased by at least 30% in Canada [3] over the interval between (1941–1945) and (1976–1980). Because the genetics of MS seems unlikely to have changed in such a short time, this increase is almost certainly due to a greater environmental exposure of the population (see below – also see Figure S1).

Establishing Plausible Parameter Ranges

11. From Equations #1 – #4 in (6) above, it is apparent that neither (c) nor (d) ultimately depend upon the value of (R). Thus,

$$\lim_{a \to \infty}\{1 - e^{-R(a - \lambda m - \lambda)}\} = \lim_{a \to \infty}\{1 - e^{-(a - \lambda m - \lambda)}\} = 1$$

And as the exposure (a) increases, (Zm) and (Zw) approach the values of (c) and (d) respectively, regardless of the value of (R). Nevertheless, the estimate of (d), is impacted by the

value (R). When the value of $(R = 1)$ is chosen then, from (7) and (9) above:

$$1.037 *(b / g_1)\} \leq d \leq 1.944 *(b / g_1)$$

$$0.149 * (b / g_2) \leq c \leq 0.369 *(b / g_2)$$

$$3.5 * (g_2 / g_1) \leq (d / c) \leq 10.6 * (g_2 / g_1)$$

When $(R \rightarrow \infty)$: $3.3 * (g_2 / g_1) \leq (d / c) \leq 8.8 * (g_2 / g_1)$

12. From (1) and (4) of *Supplemental Material #1*, combining the relationships:

$$g = Ag_1 + (1 - A)g_2 \ ; \quad \text{and:} \quad g_1 = Bg_2 + (1 - B)$$

leads to: $g_2 = \{g - (1 - B)A\} / \{1 - (1 - B)A)\}$

Using the possible range for (B) from the *Supplemental Material #1* (gender partition), together with the estimate of: $g \geq 0.84$; yields a range of possibilities:

from: $g_1 \geq 0.90$; and: $g_2 \geq 0.69$

to: $g_1 \geq 0.85$; and: $g_2 \geq 0.74$

so that, at the value of $(g = 0.84)$: $0.77 \leq (g_2 / g_1) \leq 0.87$

Of course (g) could be higher than this, in which case: $(g_2 / g_1) < 1$

and, thus, when: $R \geq 1$; then: $2.7 \leq (d / c) \leq 10.6$

When: $R = 0.1$; then: $(d / c) \geq 2.9$

Consequently, under any conceivable circumstance, at a minimum:

$$P(MS \mid E, G, F) \geq 2.7 * P(MS \mid E, G, M)$$

and, as a result, the distribution of $\{x_i\}$ in the set (X) must also be bimodal based on gender [30].

Also, using the limits for (g_1), (g_2), and (C) from above when $(R = 1)$, yields:

$$1.15(b) \leq d \leq 2.29(b)$$

and: $$0.20(b) \leq c \leq 0.53(b)$$

These relationships are illustrated in Figure S1. Moreover, given these limits, and given the possible range for (b) provided in the Main Text, therefore, it must be the case that:

$$P(MS \mid E, G, F) \leq 0.4 * 2.29 = 0.92$$

and: $$P(MS \mid E, G, M) \leq 0.4 * 0.53 = 0.21$$

Gender-Specific Hazard Rates and Thresholds

13. When $(R = 1)$, then re-arrangement of Equations #1 and #2 from (6) above yields:

$$(Zw_2 - d)/d = -e^{-(x1+1-\lambda m-\lambda)} = -e^{-(x1+1-\lambda m)} * e^{\lambda}$$

and: $$(Zm_2 - c)/c = -e^{-(x1+1-\lambda m)}$$

Dividing these two Equations yields:

$$(c/d)\{(Zw_2 - d)/(Zm_2 - c)\} = e^{\lambda}$$

so that: $$\lambda = \{\ln[(c/d)(Zw_2 - d)/(Zm_2 - c)]\}$$

Similarly, in this circumstance:

$$\lambda = \{\ln[(c/d)(Zw_1 - d)/(Zm_1 - c)]\}$$

Consequently, from (9) above:

$$c = (Zm_2)\{1 - [P(M \mid MS)_1 \,/\, P(M \mid MS)_2]*C*e^{-1}\} \,/\, (1 - e^{-1})$$

and: $$d = (Zw_2)\{1 - [P(F \mid MS)_1 \,/\, P(F \mid MS)_2]*C*e^{-1}\} \,/\, (1 - e^{-1})$$

Defining (K_M) and (K_F) to be:

$$K_M = [P(M \mid MS)_1 \,/\, P(M \mid MS)_2]*C*e^{-1} = 0.337*C$$

$$K_F = [P(F \mid MS)_1 \,/\, P(F \mid MS)_2]*C*e^{-1} = 0.483*C$$

Then the Equation: $$\lambda = \{\ln[(c/d)(Zw_2 - d)/(Zm_2 - c)]\}$$

can be rewritten as:

$$\lambda = \ln\{\{(1-K_M)*[1-(1-K_F)/(1-e^{-1})]\} / \{(1-K_F)*[1-(1-K_M)/(1-e^{-1})]\}\}$$

Using the range for (C) from above, it follows that:

$$0.10 < \lambda < 4.67 \;\; ; \quad \text{and, in fact:} \quad \forall(C>0) : \lambda > 0$$

Notably, these estimates do not depend upon the values of (c), (d), (Zw_2), or (Zm_2).

14. For susceptible women let the terms (a_1^{app}), (λ_w^{app}), (λ^{app}), and: (R^{app}) be defined at the exposure level of (a_1) such that: $$a_1^{app} = R*a_1$$

$$a_1^{app} - \lambda_w = a_1 - \lambda_w^{app}$$

and: $$\lambda^{app} = \lambda_w^{app} - \lambda_m$$

where: $$R^{app} = 1$$

From these definitions it follows that:

$$a_1^{app} - a_1 = (R-1)*a_1 = \lambda_w - \lambda_w^{app}$$

or: $$\lambda_w^{app} = \lambda_w - (R-1)*a_1$$

and, thus: $$\lambda^{app} = (1-R)*a_1 + \lambda_w - \lambda_m = (1-R)*a_1 + \lambda$$

This transformation from (λ_w) to (λ_w^{app}) is sufficient to define uniquely an exponential failure curve, which passes through the points (λ_w^{app}, $Zw = 0$) and (a_1 , $Zw = Zw_1$), which plateaus at ($Zw = d$), and which has a hazard-rate where ($R^{app} = 1$). However, unless it is actually the case that ($R = 1$), this curve will not pass through the point (a_2 , $Zw = Zw_2$) .

Nevertheless, from (13) above and using this transformation, it follows that:

$$\lambda^{app} = \ln\{(c / d)(Zw_1 - d) / (Zm_1 - c)\} > 0$$

in which case: $\lambda^{app} = (1 - R) * a_1 + \lambda > 0$

Because, by definition: $a_1 \geq 0$

Therefore: $\forall (R \geq 1) : \lambda > 0$

Thus, it follows from Assumption (F) that susceptible men must have a greater hazard-rate, a lower threshold, or both in comparison to susceptible women (see Figure S1).

Gender-Specific Exposures

15. For women, because:

$$P(MS \mid E, G, M)_2 = P(MS \mid E, G, M) = c$$

therefore: $Zm_2 = P(MS, E \mid G, M)_2 = P(E \mid G, M)_2 * c$

so that: $P(E \mid G, M)_2 = Zm_2 / c$

From (9) above: $(Zm_2) / c = (1 - e^{-1}) / \{1 - [P(M \mid MS)_1 / P(M \mid MS)_2] * C * e^{-1}\}$

so that: $0.72 \leq P(E \mid G, M)_2 = Zm_2 / c < 1$

Consequently, currently, the large majority of the susceptible men (>70%) experience an environmental exposure sufficient to produce MS in at least one susceptible person. Notably, this estimate does not depend upon the actual value of either (c) or (Zm_2). Similarly, for women, when ($R \geq 1$) :

$$(Zw_2) / d = (1 - e^{-R}) / \{1 - [P(F \mid MS)_1 / P(F \mid MS)_2] * C * e^{-R}\}$$

so that: $0.70 \leq P(E \mid G, F)_2 = Zw_2 / d < 1$

Thus, in this circumstance, the range of possible exposures seems to be quite similar in susceptible men and women. Despite this, the predicted lower bound for this possible exposure-range for women will become substantially lower than that for men when $(R \ll 1)$.

Gender Specific Susceptibility

16. From Assumption (*H*):

$$P(MS, F \mid G, IG_{MS}) / P(MS, F \mid G) \approx P(MS \mid G, IG_{MS}) / P(MS \mid G)$$

and: $$P(MS, M \mid G, IG_{MS}) / P(MS, M \mid G) \approx P(MS \mid G, IG_{MS}) / P(MS \mid G)$$

So that, from (10 – Main Text): $P(MS \mid G, IG_{MS}) / P(MS \mid G) \leq (0.081/0.059) = 1.373$

Following the logic from (8) and (10) in the Main Text:

$$P(G,F) \geq g_1^2 * P(MS,F)/t \quad ; \quad \text{and:} \quad P(G,M) \geq g_2^2 * P(MS,M)/s$$

also: $$P(G,F) \leq 1.373 * P(MS,F)/t \quad ; \quad \text{and:} \quad P(G,M) \leq 1.373 * P(MS,M)/s$$

From (5) above and from Table S2 (*Supplemental Material #1*):

$$t = 1.340b \quad ; \quad s = 0.265b \quad ; \quad \text{and:} \quad P(F \mid MS) = A = 0.706$$

And from (12) above, it follows that:

if: $g \geq 0.84$ then: $g_2 \geq 0.69$ and: $g_1 \geq 0.90$

Therefore: $$P(G, F) \geq (0.90)^2 * (0.706/1.340) * P(MS)/b = 0.43 * P(MS)/b$$

and: $$P(G,F) \leq 1.373 * P(MS,F)/t = 1.373 * (0.706/0.1.340) * P(MS)/b$$
$$= 0.72 * P(MS)/b$$

Therefore: $$0.43 * P(MS)/b \leq P(MS, F) \leq 0.72 * P(MS)/b$$

And similarly: $$0.53 * P(MS)/b \leq P(MS, M) \leq 1.52 * P(MS)/b$$

These ranges overlap somewhat. Nevertheless, if anything, men are more likely to be in the genetically susceptible subset (*G*) than women. This is especially the case if the estimate of (*g*) were too low. Thus, when $\{g > 0.9\}$, the two ranges no longer overlap.

Table S3. Additional Definitions for *Supplemental Material #2*

Time Period 1	=	*1941-1945* ; indicated by the subscript 1
Time Period 2	=	*1976-1980* ; indicated by the subscript 2
C	=	$P(MS)_1 / P(MS)_2$; Ratio of $P(MS)$ in the 1st time period to that in the 2nd
Zw	=	$P(MS,E \mid G,F) \approx P(MS \mid G,F)$; by Assumption D<br>probability of developing MS in "genetically susceptible" women
Zm	=	$P(MS,E \mid G,M) \approx P(MS \mid G,M)$; by Assumption D<br>probability of developing MS in "genetically susceptible" men
E	=	set of all those environmental experiences, which are sufficient to produce MS in at least one "genetically susceptible" individual
u	=	$P(E)$; actual units of exposure
a	=	transformed exposure level ; arbitrary units
b	=	$P(MS \mid IG_{MS})$ – See Main Text
$a_2 - a_1$	=	Transformed exposure-difference between the 2nd and 1st time-period – defined as "1 unit" of exposure
$P(E)_2$	=	Probability of sufficient exposure during the 2nd time-period
$h(u)$, $g(u)$	=	hazard-rate functions for developing MS in susceptible men $\{h(u)\}$ and women $\{g(u)\}$
R	=	proportionality constant such that: $g(u) = R*h(u)$
λm , λw	=	Exposure-threshold necessary to produce MS in "genetically susceptible" men (λm) and women (λw)
λ	=	$\lambda w - \lambda m$; difference in exposure-threshold between susceptible women and men
c, d	=	maximum probability of MS in "genetically susceptible" men (c) and women (d).<br>Thus: $c = P(MS \mid G,E,M)$; $d = P(MS \mid G,E,F)$

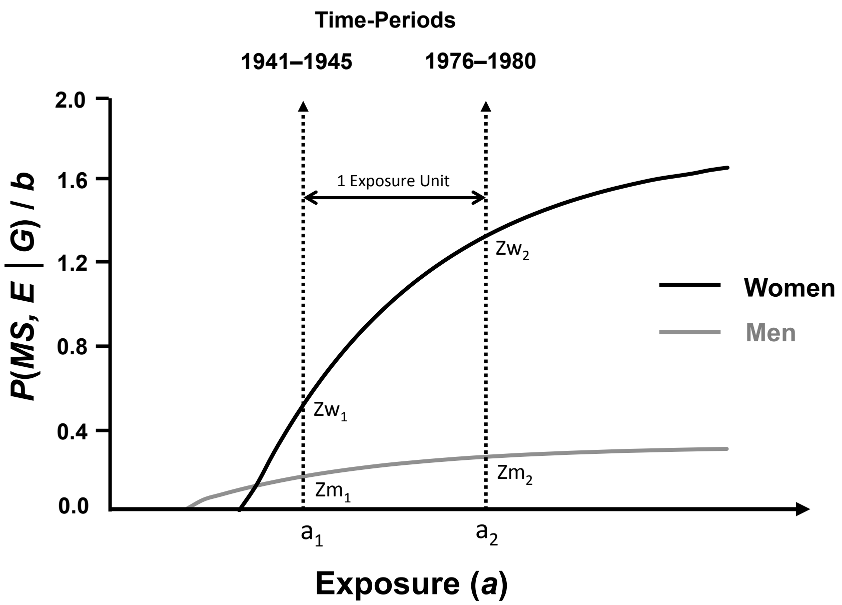

Figure S1. Response curves for the likelihood of getting MS following a sufficient environmental exposure for genetically susceptible men and women in Canada. Data taken from the study of Orton and colleagues [40]. The probability of getting MS – i.e., $P(MS, E \mid G)$ – is shown on the y-axis in units of (b). The exposure for the population is shown on the x-axis in transformed units of (a). Labels for terms $\{Zw = P(MS, E \mid G, F)\}$ and $\{Zm = P(MS, E \mid G, M)\}$ are provided at each time-point. One "exposure unit" is defined arbitrarily as $(a_2 - a_1)$. In the *Supplemental Material #2*, possible ranges for the different parameters are derived.

For illustrative purposes, values near the mid-points of the ranges have been chosen and the plots represented are for the conditions: $(C = 0.5\ ;\ \text{and: } R = 1)$. Also, proportional hazard has been assumed for men and women (see Assumption F). Changes to the value of (C) will only alter the units of the y-axis. Changes to (R) will alter how quickly the curves reach their plateau and, thus, will impact the threshold for men relative to women − if men have a greater hazard (i.e., if: $R < 1$) their threshold will move closer to women's. If the hazard is not proportionate, the points (Zw_1 *and* Zw_2) will remain the same although, for women, the path prior to, between, and subsequent to these two points would be undefined. Men would still follow the curve shown. Nevertheless, women (under any circumstance) still have an increased response (relative to men) to the changes in environmental exposure, which have taken place between the two time-periods. (*Adapted with permission from Goodin DS. PLoS One 2009;4(2):e4565*)

Unimodal *vs.* Bimodal Distributions

Supplemental Material #3

1. Asymmetric unimodal distribution – the log-normal distribution…….. p. 2
2. The Nature of Genetic Susceptibility ………………..………………... p. 3
3. Table S4 – Additional Definitions …………………...………………... p. 6
4. Figure 2S A – Different log-normal distributions …………………….. p. 7
5. Figure 2S B – A log-normal distribution with a 92% / 8% split …..….... p. 8

Asymmetric Unimodal Probability Distributions

The log-normal distribution is an asymmetric probability distribution that has considerable theoretical appeal, particularly in the setting of complex diseases such as MS, which are known to be associated with multiple genetic risk factors [5–9]. Thus, in MS, over 110 non-MHC genes have already been associated with the disease and many more are suspected of being involved [5–9]. The reason for this appeal is that, if these multiple risk factors are independent of each other and the odds are multiplicative, then (by the central limit theorem) the resulting probability distribution of the odds of MS will follow a log-normal probability density function. New definitions specific to *Supplemental Material* #3 are presented in Table S4.

Here, we will define the following six quantities:

1. μ = mean of the log-transformed probability distribution for MS odds
2. σ = standard deviation of the log-transformed probability distribution.
3. m = the mean of the non-transformed probability distribution
4. v = the variance of the non-transformed probability distribution
5. t = mean of the non-transformed probability distribution for the odds in those individuals with probabilities of MS at or above $P(MS)$.
6. ω = odds of developing MS for an individual in the population. $\{\text{range: } (\omega = 0) \text{ to: } (\omega = +\infty)\}$

For the log-normal distribution these variables are related such that:

$$\mu = \ln\{m / \sqrt{(1 + v / m^2)}\}$$

and:

$$\sigma = \sqrt{\ln(1 + v / m^2)}$$

Aligning all distributions at $(\mu = 0)$, the 1st equation can be rearranged to yield:

$$(m^2)^2 - m^2 - v = 0$$

or:

$$m^2 = 1/2 \pm (\sqrt{1 + 4v})/2$$

This permits the calculation of (σ) and (m) at various values of (v). This, in turn, permits the calculation of the cumulative distribution function (*cdf*) of the log-normal distribution from ($\omega = 0$) up to the point $\{\omega = m = P(MS)\}$ – see Figure 2S A – which provides a measure of the degree of asymmetry for the distribution.

In order to achieve the minimum permissible degree of asymmetry, which (as discussed in the Main Text) is a split of 8.5% of the population being in the (G) subset 91.5% being in the (G_T–) subset, requires that ($\sigma = 2.74$). At this standard deviation, the mean of the distribution (t) for those individuals who have an odds of developing MS at or above $\{m = P(MS)\}$ is more than 4.6 fold less than the odds at the minimum mean for the (G) subset (see Figure 2S B).

Or, restated: $P(MS \mid G) > 4.6^*t$

Moreover, a similar degree of separation persists at even more markedly asymmetric splits (see legend, Figure 2S B). Thus, this marked disparity (i.e., separation) between $\{(t)$ and $P(MS \mid G)\}$ indicates that the (G) subset cannot be accounted for by ascribing it to merely being the tail of a log-normal distribution (see Figure 2S B). Consequently, the population (P_0) must be, at least, bimodal with respect to the likelihood that MS will develop in individual members [30].

The Nature of Genetic Susceptibility

It might be argued that the presence of genetic susceptibility in only a small portion of the population (i.e., a bimodal distribution) would lead to either linkage or strong interactions being detectable (which, so far, have not been observed). However, this argument is incorrect for disorders that involve many susceptibility alleles. For simplicity, we will consider all susceptibility to be in (G), only alleles that increase the likelihood of being in the (G) subset (indicated by capitol letters), and that linkage between the high-susceptibility (G) subset and the allele (A) exists when:

$$P(G \mid A) = 1$$

In addition, again for simplicity, we will consider that a strong interaction between alleles (A) and (B) exists when:

$$P(G \mid A,B) = 0$$

Clearly, weaker linkages and interactions may well exist but, for illustrative purposes here, it is sufficient to consider only the strongest linkages and the strongest interactions (i.e., present or absent – as defined above).

When there is only one susceptibility allele, there is only linkage. When there are two susceptibility alleles, then there is either linkage or a strong interaction.

Thus, either: $P(G \mid A) = 1$; and: $P(G \mid B) = 1$

or: $P(G \mid A,B) = 0$ (this is the only interaction possible)

In this situation it might be easier (with alleles that increase risk) to envision the linkage scenario although the interaction scenario is possible if either (*A*) or (*B*) create an imbalance in some system for which, when they occur together, balance is restored.

With four susceptibility alleles, consider the circumstance in which only the genotypes (*A,B,D*) and (*A,C,D*) lead to susceptibility. Again, there is no linkage although there are strong interactions {e.g., $P(G|B,C,D) = 0$; and: $P(G|A,B,C) = 0$}. However, these interactions are of a higher order and the number of interaction terms that need to be considered in order to discover these relationships has increased to 11. By contrast, if the genotype (*A,B,C,D*) is also in (*G*), many of the strong interactions will, likely, disappear. This scenario is, again, easier to rationalize (with alleles that increase risk). Thus, perhaps, there are three metabolic pathways, which need to be involved for an individual to be susceptible, and that both (*B*) and (*C*) represent redundant lesions in the same pathway. If so, then it is also easy to imagine that the MS is no more likely for the (*A,B,C,D*) genotype than it is for either of the other two susceptible genotypes (i.e., the odds of MS is not increased by the addition of the 4th risk allele). The possible (perhaps likely) complexities increase dramatically with each additional risk allele. For example, with five alleles, there are 26 possible interactions; with six alleles, there are 57; and so forth.

For MS, more than 110 genomic regions have been identified as being associated with MS – i.e., these are loci that harbor some number of alleles, which either alone or in combination with other alleles, increase or decrease the likelihood of getting MS compared alternative alleles in the same region [6,7]. Therefore, in MS, the detection of linkage or strong interaction are not expected. For example, although MS cases have slightly more of these loci in a "susceptible state" compared to controls, there is considerable overlap. Thus, of the first 95 MS-associated SNPs identified in the WTCCC dataset [6,7], in MS cases, 32% of these risk alleles are homozygous compared to only 30% in controls. By contrast, controls have no copies of 31% of these risk alleles compared to only 29% in MS patients. An equal percent in cases and controls are heterozygous. Because of the large numbers of cases and controls in this dataset, this difference is statistically significant. However, the standard deviations for each of these estimates are more than 7 fold greater than any of the observed differences.

From these data, and because, likely, the large majority of MS patients are in the (*G*) subset (*Supplemental Material #1*), it seems clear that high-susceptibility requires

only a subset of these 110 risk alleles. Moreover, this number of 110 risk alleles in the genome is necessarily a minimum estimate and, in MS, there are many more loci suspected of being involved. Regardless of the exact organization, however, it must be the case that an individual belongs to the (*G*) subset only if they possess an appropriate combination of some number of risk alleles, which collectively give the individual an average likelihood (or greater) of developing MS. Moreover, there must be some average number of such alleles that need to be present for a person to be in the subset (*G*). Naturally, not all of these highly-susceptible combinations will have the same number of alleles and not every combination of a certain number will cause a person to be in the subset (*G*). Nevertheless, if an individual possesses such a highly-susceptible combination, it is not at all clear that, in every case, having more loci in "susceptible allelic states" will necessarily affect the resulting likelihood of MS (an essential component of the log-normal model). For example, in the last scenario for the four risk alleles (described and discussed above), the addition of the 4th risk allele (potentially) has no impact on the odds of MS.

Moreover, the number of possible combinations of risk alleles is almost certainly huge. For example, if susceptible genotypes in (*G*) consisted of combinations of somewhere between 10 and 90 of these risk alleles, the total number of possible combinations exceeds (by many, many orders of magnitude) the number of people who have ever lived. Even if this range were substantially narrower (even to a fixed number within this range) and/or if only a tiny fraction of the possible combinations lead to membership in the subset (*G*), the situation is unchanged. The number of potential combinations is just too large. Thus, it seems very likely that the particular combination of these 110 risk alleles in almost every MS patient will be unique. Empiracally, this seems to be case, at least for the vast large majority of MS patients. Thus, considering the first 95 MS-associated SNPs identified in the WTCCC data set [6,7], 105 genotypes (at these SNP locations) were identical in, at least, 1 pair of MS cases. Nevertheless, for all of the remaining 10,643 MS cases in this dataset, their genotypes at these locations were unique. Moreover, none of these apparently duplicated genotypes bore any obvious resemblance to each other (sharing identity at only 43 of the 95 SNPs, on average). Under such circumstances, almost certainly, there will be no linkage and no strong interactions, even if the population (P_0) is bimodal.

Table S4. Additional Definitions for *Supplemental Material #3*

μ	=	mean of the (transformed) log-normal distribution
σ	=	standard deviation of the (transformed) log-normal distribution
m	=	odds of MS at the mean for the (non-transformed) log-normal distribution
v	=	variance of the (non-transformed) log-normal distribution
t	=	mean of the (non-transformed) log-normal distribution for individuals who have an odds of MS at or above $P(MS)$
ω	=	odds of developing MS for an individual in the population (P_0).
$P(MS)$	=	m
$P(MS \mid G)$	=	odds of MS at the mean for the (G) subset – see Main Text

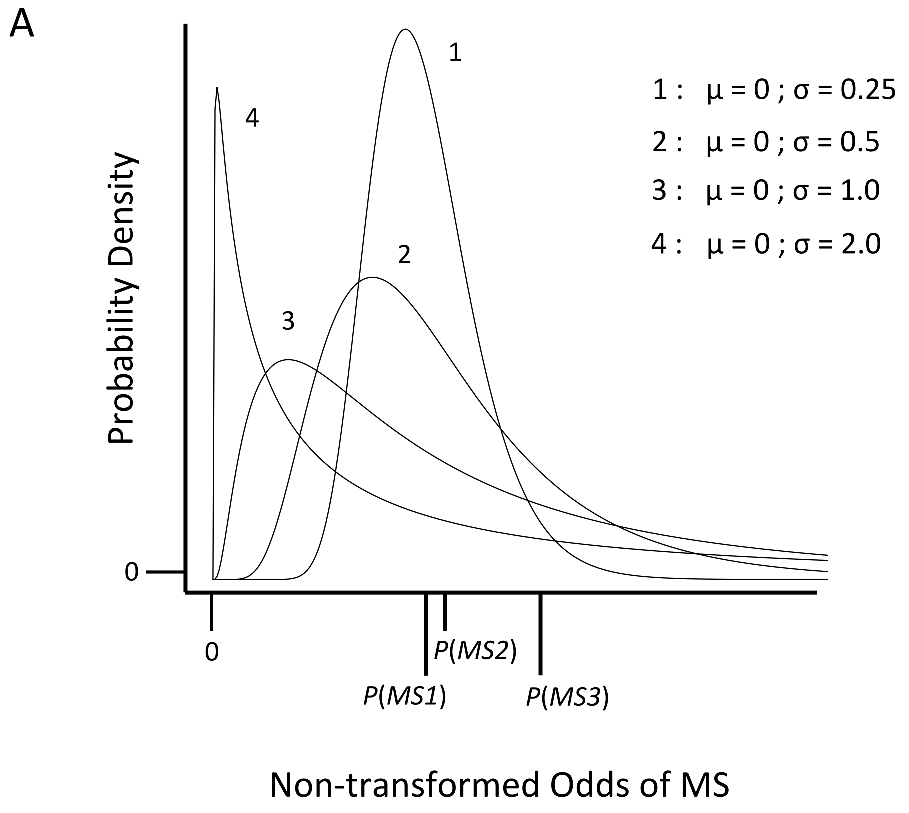

Figure S2 – (A) Plot of several different hypothetical log-normal probability distributions (not transformed) for the odds of MS given the conditional probabilities for MS of the different genotypes in the (P_0) population. The mean (μ) for the normal distribution (i.e., after log-transformation of these odds) has been set to 0 in all cases (i.e., non-transformed odds = 1). What differs between the hypothetical curves is the value of the standard deviation (σ) for this normal distribution. This alignment makes the odds of MS at *P*(*MS*) – the mean probability of MS for (P_0) population – move to the right as the value of (σ) increases and as the distributions become increasingly skewed. *P*(*MS1*) represents the odds of MS at this mean for distribution #1. Similarly, *P*(*MS2*) and *P*(*MS3*) represent the odds of MS at this mean for distributions #2 and #3 respectively. *P*(*MS4*) has not been depicted in the Figure because it lies off scale to the right {at a location = (7.2)**P*(*MS1*)}. This convention has been adopted for illustrative purposes only. Naturally, in actuality, it is {*P*(*MS*)} that remains fixed and the non-transformed odds at the mean (μ), which moves closer to zero with increasing asymmetry of the distribution. Moreover, because the value of *P*(*MS*) is so small in the population (≤ 0.005), there is little difference between the risk (probability) of MS and the odds of MS.

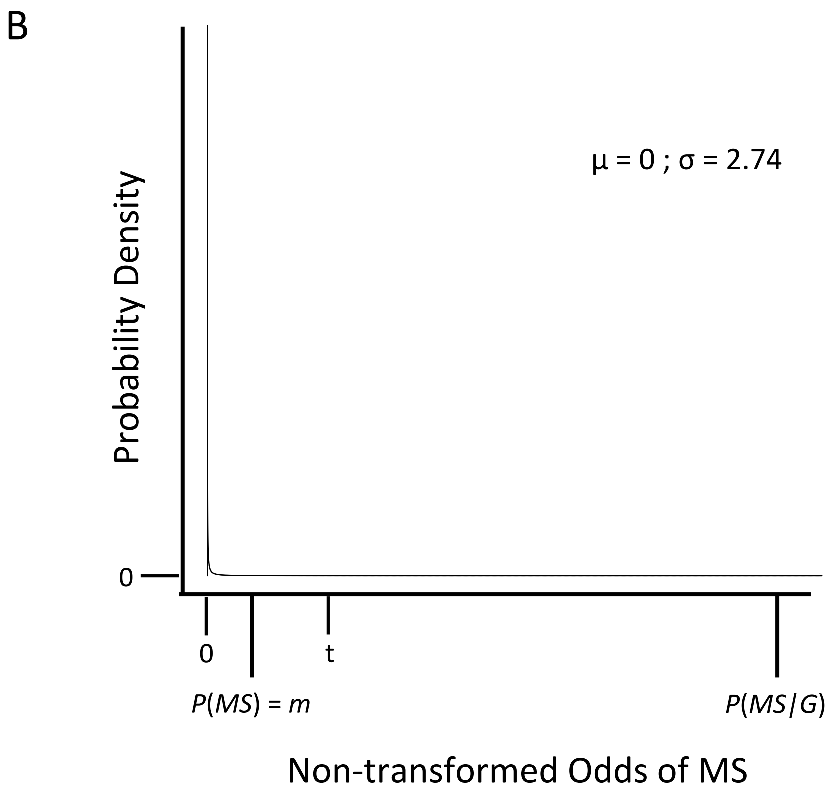

Figure S2 – (B) Plot of a hypothetical log-normal probability distributions (not transformed) for the odds of MS given the conditional probabilities for MS of the different individual genotypes in the population. The mean (μ) of the normal distribution (i.e., after log-transformation of these odds) has again been set to 0. The value of the standard deviation (σ) for this normal distribution (2.74) has been chosen because, at this value, the distribution is asymmetric such that 91.5% of the individuals have and odds of MS less than the odds at the mean of the non-transformed distribution $\{m = P(MS)\}$ and 8.5% have an odds at or above this value. On the x-axis, the point (t) represents the odds at mean of the non-transformed distribution for those individuals who have an odds of MS at or above $\{P(MS) = m\}$. The point $P(MS|G)$ represents the minimum odds of MS at the mean probability of MS for the subset (G) – see Main Text. The odds at the minimum $P(MS|G)$ are more than 4.6 times the odds at (t) and this kind of disparity persists for even more asymmetric splits. Thus, at the minimum of $\{P(G|MS) = 43\%\}$ – see Section 9; *Supplemental Material #1* – then the $\{P(G) \geq 0.0018\}$ – see Section 8; *Main Text* – and $\{P(MS|G) \geq 3.4*(t)\}$. Even considering a huge split of $\{P(G) = 10^{-14}\}$, it is still the case that $\{P(MS|G) \geq 3.1*(t)\}$. Thus, under no circumstance can the odds at (t) even approach the odds at $P(MS|G)$ and, consequently, $P(G)$ cannot be explained as the tail of a log-normal distribution.
